# Thermocapillarity in Microfluidics—A Review

**DOI:** 10.3390/mi7010013

**Published:** 2016-01-21

**Authors:** Alireza Karbalaei, Ranganathan Kumar, Hyoung Jin Cho

**Affiliations:** Department of Mechanical and Aerospace Engineering, University of Central Florida, Orlando, FL 32816, USA; akarbalaei@knights.ucf.edu (A.K.); ranganathan.kumar@ucf.edu (R.K.)

**Keywords:** thermocapillary, microfluidic, actuation, MEMS devices, evaporation, instability, droplet, bubble

## Abstract

This paper reviews the past and recent studies on thermocapillarity in relation to microfluidics. The role of thermocapillarity as the change of surface tension due to temperature gradient in developing Marangoni flow in liquid films and conclusively bubble and drop actuation is discussed. The thermocapillary-driven mass transfer (the so-called Benard-Marangoni effect) can be observed in liquid films, reservoirs, bubbles and droplets that are subject to the temperature gradient. Since the contribution of a surface tension-driven flow becomes more prominent when the scale becomes smaller as compared to a pressure-driven flow, microfluidic applications based on thermocapillary effect are gaining attentions recently. The effect of thermocapillarity on the flow pattern inside liquid films is the initial focus of this review. Analysis of the relation between evaporation and thermocapillary instability approves the effect of Marangoni flow on flow field inside the drop and its evaporation rate. The effect of thermocapillary on producing Marangoni flow inside drops and liquid films, leads to actuation of drops and bubbles due to the drag at the interface, mass conservation, and also gravity and buoyancy in vertical motion. This motion can happen inside microchannels with a closed multiphase medium, on the solid substrate as in solid/liquid interaction, or on top of a carrier liquid film in open microfluidic systems. Various thermocapillary-based microfluidic devices have been proposed and developed for different purposes such as actuation, sensing, trapping, sorting, mixing, chemical reaction, and biological assays throughout the years. A list of the thermocapillary based microfluidic devices along with their characteristics, configurations, limitations, and improvements are presented in this review.

## 1. Introduction

As the application areas of microfluidics expand from the traditional mechanical engineering domain to more complex chemical and biological systems, various flow patterns and phenomena that could be favorably used at the reduced scale have been actively studied. At the microscale, the effect of capillary and van der Waals forces become more dominant over convection and other long range body forces, making the fluid actuation by means of capillarity attractive. The capillary action based on the change of surface or interfacial tension of liquid in contact with solid could be manipulated with electric field, magnetic field, thermal gradient as well as chemical concentration gradient. Various terms such as electrocapillary, electrotaxis, magnetocapillary, magnetotaxis, thermocapillary, thermotaxis, chemocapillary and chemotaxis have been used to describe these observations and experiments. 

Among those, electrocapillary effect has been widely used due to its applicability to straightforward device designs and operations; two capacitive electrodes acting on a conductive liquid droplet. Although thermocapillarity has been studied for a longer period of time and has been more universal since the change of surface tension under temperature gradient is applicable to any liquids including non-conductive, its practical implementations into microfluidic devices have been largely hampered by evaporation, hysteresis, and pinning associated with heating.

**Figure 1:**
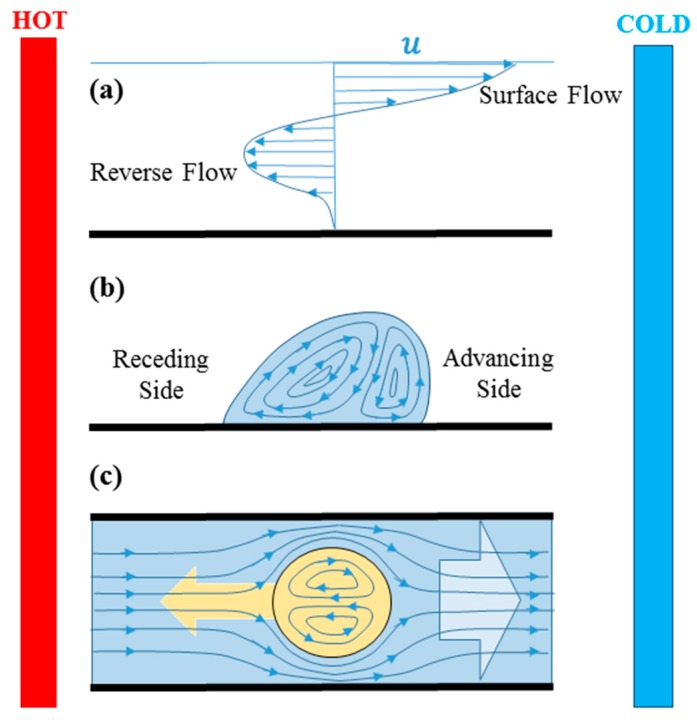
Surface Marangoni flow induced due to surface temperature gradient from hot to cold region. The flow propagates inside due to drag and reverse flow forms to hold mass conservation (**a**). Droplet on a solid surface with temperature gradient leans toward the cold region due to higher surface tension at the inception of movement and Marangoni circulation forms inside it (**b**). In the case of channel flows, the carrier liquid obeys a bulk flow from hot to cold region due to surface tension forces and the confined droplet moves to the opposite direction (toward the hot region) to conserve the mass (**c**).

There are two basic modes of flow generated by surface tension gradient. Temperature gradients perpendicular to the liquid layer produce Marangoni instability and construct Benard-Marangoni circulations inside the liquid layer. Temperature gradients tangential to the liquid surface on the other hand produce surface tension gradient along the surface of the liquid and induce surface flow from low to high surface tension regions. As shown in Figure 1a, the surface flow diffuses in depth due to continuity of shear stress, and eventually a reverse flow forms in deeper areas to conserve the mass. Marangoni circulations form inside drops on solid substrates due to leaning of the drops to the colder side and the tendency of liquid layers to move to the peak of the drops which is colder and has higher surface tension (Figure 1b).

Thermocapillary-induced bulk flow in microchannels leads to the movement of any immersed or floating object, including bubbles and droplets (Figure 1c). Drag at the interface of the dispersed and continuous phases transfers the bulk motion and induces circulation inside the bubble or droplet and mass conservation moves the immersed bubble/drop to the opposite direction of the bulk flow. Researchers have proved that thermocapillarity can produce flow circulations (Marangoni instabilities), affect the evaporation physics, and most importantly actuate fluid elements, which becomes more significant when the scale becomes small. Various computational methods have been used to capture flow patterns inside bubbles, droplets and liquid films subject to heat transfer. Thermocapillary-based valves, pumps, and mixers have been proposed to control fluid elements and perform biological and chemical experiments on the controlled samples.

Comprehensive reviews on microfluidics have been given by Squires and Quake [1], Stone *et al.* [2], Fair [3], Darhuber and Troian [4], and Baroud *et al.* [5]; but the topical review on thermocapillary in microfluidics has not been presented by any, to our knowledge. This paper reviews past and recent achievements in thermocapillary studies in relation to microfluidics. The theoretical framework of thermocapillarity in microfluidics has been presented in Section 2. Thermocapillary-induced instabilities are explained in Section 3 followed by evaporative instabilities and thermocapillary effect on evaporation of drops in Section 4. Thermocapillary effect on spreading, migration, coalescence, and mixing of bubbles and droplets are the subjects of the review in Section 5. Finally, proposed thermocapillary-based microfluidic devices for different applications are reviewed in Section 6.

## 2. Theory

Fluid motion is governed by the Navier-Stokes equations [6,7,8] which relate the temporal and spatial change of the fluid velocity field to the shear and normal stresses and the body forces. The only body force in the absence of electrical and magnetic fields is gravity. Surface tension—on the other hand—only acts on the free surface of the fluid and hence appears only in boundary conditions which aim to balance normal and tangential stresses on the free surface (Equations (1) and (2), respectively):

(1) $$\hat{n}\cdot \overset{=}{T}\cdot \hat{n}=\sigma \left(\vec{\nabla }\cdot \hat{n}\right)$$

(2) $$\hat{n}\cdot \overset{=}{T}\cdot \hat{t}=\vec{\nabla \sigma }\cdot \hat{t}$$

**Figure 2:**
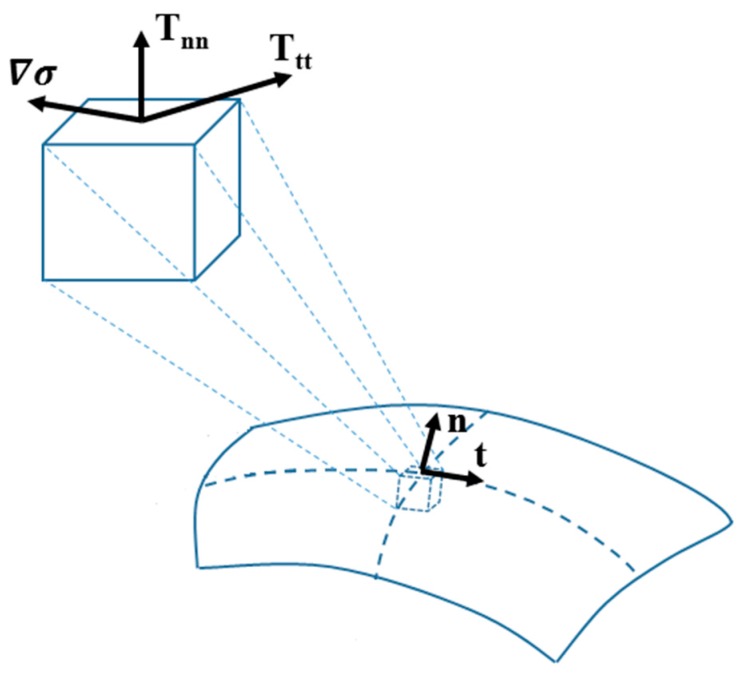
Components of the stress tensor, surface tension gradient, and normal and tangential unit vectors on a fluid surface element.

$\overset{=}{T}$ in these two equations is the stress tensor, σ is the surface tension, and $\hat{n}$ and $\hat{t}$ are unit normal and tangential vectors to the fluid surface, respectively (Figure 2).

These two equations state the following:

- The normal component of the hydrodynamic stresses must balance with the portion of surface tension due to curvature (Equation (1)). This equation is called Laplace equation in the absence of viscosity.
- The tangential component of the hydrodynamic stresses must balance the tangential portion of the surface tension gradient (Equation (2)).

In the process of normalizing the governing equation and boundary conditions, nondimensional parameters are frequently used to simplify the problems. The list of these nondimensional numbers is presented in Table 1. In most cases in microfluidics, capillarity and viscosity are the two major effects on the fluid element. For example, capillary number of one declares that viscous and capillary forces are of the same importance. By writing the force balance between gravity and capillarity on the triple line of a forming meniscus, one can define capillary length as $l_{c}=\sqrt{\sigma /\rho g}$. For the fluid flow problems with characteristic length smaller than the capillary length the capillary forces become dominant.

**Table 1:** List of the nondimensional numbers in analysis of thermocapillary fluid flows.

Number	Symbol	Definition
Capillary Number	Ca	$\frac{\mathrm{Viscosity}}{\mathrm{Capillarity}}$
Marangoni Number	Ma	$\frac{\mathrm{Thermocapillarity}}{\mathrm{Viscosity}}$
Reynold Number	Re	$\frac{\mathrm{Inertia}}{\mathrm{Viscosity}}$
Weber Number	We	$\frac{\mathrm{Inertia}}{\mathrm{Capillarity}}$
Bond Number	Bo	$\frac{\mathrm{Gravity}}{\mathrm{Capillarity}}$
Ohnesorge Number	Oh	WeRe≡ViscosityInertia⋅Capillarity
Froude Number	Fr	$\frac{\mathrm{Inertia}}{\mathrm{Gravity}}$
Biot Number	Bi	$\frac{\mathrm{Convective Heat Transfer}\left(\mathrm{liquid}\right)}{\mathrm{Conductive Heat Transfer}\left(\mathrm{Solid}\right)}$
Nusselt Number *	Nu	$\frac{\mathrm{Convective Heat Transfer}}{\mathrm{Conductive Heat Transfer}}$
Prandtl Number	Pr	$\frac{\mathrm{Viscous Diffusion Rate}}{\mathrm{Thermal Diffusion Rate}}$
Peclet Number *	Pe	$\frac{\mathrm{Convective Heat Transfer}}{\mathrm{Diffusive Heat Transfer}}$

Note: * These numbers can also be defined based on mass transfer depending on the application.

A set of simplifying assumptions are made under the name of lubrication theory in problems where one of the length scales is negligible compared to the other two. It is convenient to assume that the velocity gradient in the directions of the large length scales is one order of magnitude smaller than that in the small length scale direction and hence can be ignored. The result of this assumption will be zero pressure gradient in the small length scale direction.

The migration of fluids due to the change of surface tension was first noted by Thomson [9] and the subsequent theoretical development was done by Marangoni [10]. The mass transfer due to a change of surface tension arising from a temperature gradient was specifically coined as Benard-Marangoni effect [11]. In this review, the abbreviated term, “Marangoni effect” will be used to explain the same.

Surface tension of a liquid is dependent on its surrounding scalar field such as electrical and temperature fields as well as its chemical composition. For the relation between surface tension and temperature, Eotvos suggested Equation (3) in analogy to the gas law [12].

(3) $$\sigma {\left(M/\rho \right)}^{2/3}=K\left(T_{c}-T\right)$$

**Figure 3:**
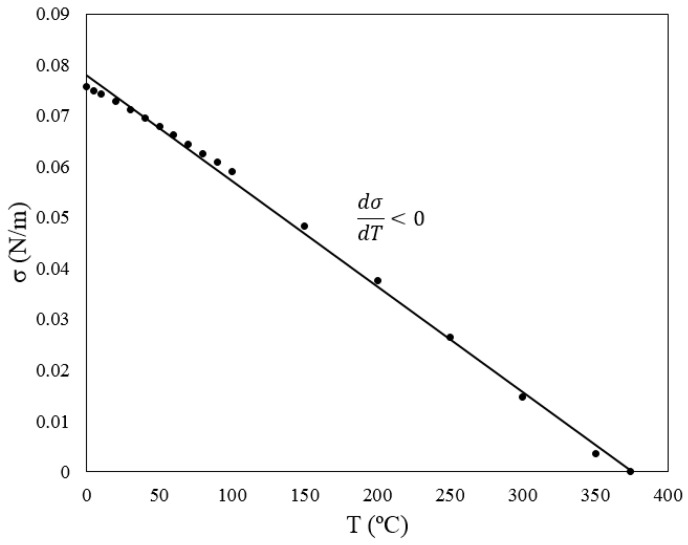
Surface tension *versus* temperature for water in contact with air.

In this equation, σ, *M*, and ρ, are the surface tension, molecular weight, and density of the liquid. *K* is a universal constant for all the non-polar liquids, and *T_c_* is the critical temperature of the liquid, *i.e.*, the end point of its phase equilibrium curve. This relationship turned out to be exact for the temperatures below the critical point of the liquid. As is shown in Figure 3 for water in contact with air, surface tension of most of the liquids is a linear function of temperature which decreases by increasing the temperature.

Several other empirical correlations have been driven between surface tension and temperature for special cases throughout the years such as the one proposed by Villers and Platten [13] for water with normal alcohols with 4 to 12 carbon atoms. They proposed a quadratic relation between surface tension and the temperature as stated in Equation (4). They also reported that surface tension of these alcohols increases with temperature up to a certain point and decreases after that.

(4) $$\sigma =AT^{2}+BT+C$$

A very comprehensive theoretical discussion was published by de Gennes [14] on the wettability under thermodynamic equilibrium. He also studied contact angle changes in irregular and random surfaces such as surfaces with parallel grooves, or weak fluctuations. The effect of different forces such as long range, van der Waals, and double layer forces, on liquid wettability has been also studied. He discussed about wetting on different surfaces such as wet or dry, or inclined surfaces, or surfaces with obstacles as well.

Another theoretical work was published by Oron *et al.* in 1997 [6] in which thermocapillarity on liquid films was expansively formulated in the form of highly nonlinear set of evolution equations. Using long wave theory, the asymptotic procedure of reduction of the original free boundary problem was presented. Their formulation showed to satisfy the lubrication theory in the case of low Reynolds number. Several test case problems which involved interfacial shear stress, surface tension, van der Waals forces, thermocapillarity, and body forces were extensively studied.

## 3. Thermocapillary Instability of Thin Films

In the instability analysis of a fluid flow -in general- the routine procedure is to feed the governing equations with a small perturbation with specific magnitude and frequency in flow characteristics such as velocity, pressure, and temperature; and obtain the solution to see under which conditions the perturbations grow and under which conditions they decay. The conditions under which the perturbations grow in time and space lead to flow instability. Disturbance in forces balance is considered a general cause of flow instabilities. Researchers have reported several kinds of flow instabilities associated with the changes in surface tension due to perturbations in the affecting flow parameters. In some cases these instabilities grow and lead to rupture in liquid films [15]. In presence of either lateral or vertical temperature gradient in the liquid films, spatial varying surface tension along with mass conservation produces circulations whose frequency and amplitude is dependent on the magnitude of the temperature gradient. Adding gravity, buoyancy, and solutocapillary to the affecting forces in multiphase multicomponent problems increases the complexity of the stability analysis. Wine tears can be brought into attention as an example of this type of behavior. In this well-known problem, films of wine climb the walls of the glass due to capillary forces until the ethanol evaporates from the film and it slips back down due to the decrease in its surface tension.

Rickett and his co-workers performed a modal and instability analysis in a medium of two fluids separated by a deformable interface with considerable scalar concentration field subject to non-uniform Marangoni forces [16]. They observed that type I and II instabilities (classified by Cross and Hohenberg [17]) by performing normal mode perturbation analysis for low wave numbers. Also, they proved that type III instability is not plausible in this case. The study of this case is applicable in the human gastric digestion. According to Cross and Hohenberg [17], type I instability happens when the perturbation temporal frequency of the stationary periodic instability approaches to zero and the flow is just spatially perturbed. For the cases where the real part (space-dependent term) is zero the stationary instability is of type II, and finally type III happens in cases where both of the imaginary and real parts survive. For more precise and in detail formulation readers are referred to reference [17].

According to the theoretical study by Ostrach in 1982 [18] and Davis in 1987 [19], thermocapillary instabilities of one-layer liquid films can be categorized in two different groups. For the cases that the temperature gradient is perpendicular to the liquid free surface, convective instabilities are produced in form of Marangoni convections for which there exists a purely steady basic state. This type of instability was first reported by Pearson in 1958 [20]. On the other hand, if the temperature gradient is tangential to the liquid interface, instabilities grow in the form of propagation of time-periodic hydrothermal waves which was first unraveled by Smith and Davis in 1983 [21]. Different States of each of these instabilities along with their associated critical conditions have been studied in detail using linear and nonlinear stability analysis, along with energy stability and bifurcation theories.

Cazabat *et al.* reported a phenomenon named as fingering instability in 1990 happened in spreading liquid films caused by Marangoni effect [22]. It is plausible for the liquid to move above the meniscus level under vertical temperature gradient at the wall. The balance between gravity and capillary forces under this condition leads to a combination of Marangoni convection and hydrothermal wave propagation called fingering instability. This phenomenon was also observed later by Kataoka and Trolan [23]. Years after that, the work on this subject continued by study of the instability of heated falling films by Joo *et al.* [24].

According to a report by Bowen and Tilly in 2012, a theoretical study has been done on liquid film rupture under thermocapillary stresses due to its heat transfer to the environment [25]. They applied long-wave analysis with small velocity deviations and derived a system of coupled partial differential equations whose solution is the film thickness, mean velocity, and temperature of the liquid film. From the linear stability analysis they found out that the velocity of the film is coupled with the interfacial dynamic and the relativity between temperature and velocity disturbances can enhance or delay the rupture. Liquid film rupture due to thermocapillary instabilities has been discussed in more detail by Burelbach [26]. Hydrothermal waves inside a sessile droplet have also been captured numerically on a heated substrate by Karapetsas *et al.* [27]. They used linear stability analysis in the quasi-steady state approximation and achieved results in agreement with previous experimental efforts. Researchers also studied some other aspects related to thermocapillary instabilities such as interface deformation [28,29,30], and jet actuation [31,32,33] over the years. Further information on thermocapillary instability is also available in the works published by Wanschura *et al.* [34], Schwabe and Scharmann [35,36,37], Neitzel [38,39,40], Schatz [41,42,43,44,45,46], and Oron [47,48,49,50,51,52].

## 4. Thermocapillary Effects in Evaporating Droplets

Evaporation can produce Marangoni-driven instabilities and such instabilities can also affect evaporation physics. Dynamics of evaporation of liquid films or droplets has been simulated through direct numerical simulation by Saenz *et al.* [53]. Thermal instabilities have been investigated during this process such as propagation of hydrothermal waves (HTWs). It has been found out that the Marangoni effect helps formation and propagation of these instabilities. Travelling the waves on the interfaces affects the evaporation rate and in some cases it might even lead to condensation due to capillarity. According to a theoretical study by Sultan *et al.* on the thermocapillary instability of a liquid drop on solid surface, the heat transfer because of evaporation leads to producing Marangoni stresses [54]. Linear stability analysis showed that this Marangoni effect is destabilizing while evaporation and capillarity are stabilizing the liquid. The evaporatively-induced Marangoni instability was also observed and analyzed by Kavehpour *et al.* throughout the spreading of a silicone oil volatile droplet on solid surface [55]. They divided the characteristic dynamics of spreading drops into viscous-capillary, viscous-inertia-capillary, and inertia-capillary regimes and reported that the physics of each of these regimes depend on the Ohnesorge number and the ratio of the droplet height (radius) to the capillary length. According to their report, there exist critical onset conditions for these instabilities and they can even be eliminated by controlling the physical properties of the liquid and the substrate. In another study by Buffone *et al.* the Marangoni instabilities were captured inside and at the meniscus interface of a liquid droplet inside a horizontally oriented capillary tube [56]. The interface periodic instabilities are believed to be sustained by the self-induced temperature difference at the triple line of the droplet. The flow pattern Marangoni convection inside the drop is a result of the competition between gravity and capillarity and is affected also by the interface instabilities.

**Figure 4:**
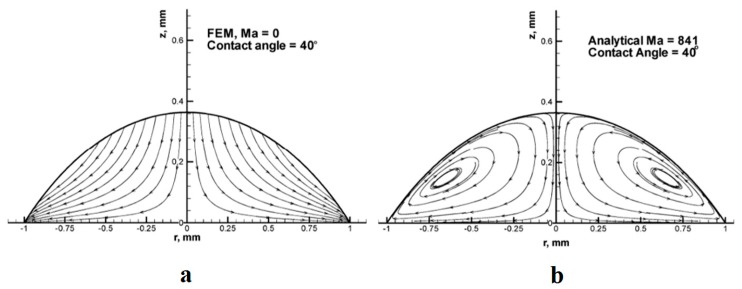
Flow pattern inside an evaporating droplet with pinned boundaries. (**a**) Without considering the Marangoni effect [60]; (**b**) with considering the Marangoni number of 841 [61]. Reprinted with permission from [60,61]. Copyright 2005, American Chemical Society.

The first effect of capillarity in relation to evaporation of liquids was observed in a common daily phenomenon named as coffee ring effect. This phenomenon that was studied both experimentally and numerically by Deegan *et al.* in 2000 [57] is described as leaving the colloids of a drying droplet in a ring shape on the solid surface after it is completely evaporated. Outward flow within the droplet due to higher capillarity at the solid/liquid interface along with droplet pinning as a geometrical constraint were reported as the reasons for this phenomenon. This flow field was simulated by Fischer [58], and also Hu and Larson [59,60,61], in separate studies and also years later the phenomenon was re-observed by Bhardwaj [62]. Hu and Larson studied the droplet evaporation with a pinned droplet on a solid substrate experimentally and theoretically [59]. They found out that droplet evaporation can be considered a quasi-steady state process under some special conditions and hence the evaporation rate turned out to be constant. According to their report, there exist a critical contact angle at which the contact line starts to recede. They also employed finite element method to simulate the flow field inside an evaporating droplet with using lubrication theory in low Reynolds and Capillary Numbers, applied it to both pinned [60] and unpinned [61] boundaries, and reported different flow pattern for each case. Figure 4 shows their numerical result of flow pattern inside an evaporating droplet with and without the Marangoni effect.

In a recent numerical study, Barash has shown that depending on the relative conductivity of substrate and the liquid droplet, not only one, but two or three vortices can form in different locations inside the evaporating droplet [63]. He graphed the number, location, and orientation of the forming vortices in a phase diagram and captured the temperature distribution inside the droplet as well. Shih and Megaridis in another numerical attempt [64] showed that flow circulation happens inside droplets due to surface tension gradient along the liquid-gas interface. This circulation decreases the drag coefficient and increases the evaporation rate of the droplet independently from Reynolds number. This finding was in contrast to Marek and Straub’s report about decaying the evaporation (and condensation) coefficients with increasing temperature [65]. They defined evaporation (condensation) coefficient as the ratio of molecules absorbed by the vapor (liquid) phase to the number of molecules emitted from (impinged to) the liquid phase. Later, Ajaev and his group developed a mathematical model for droplet evaporation on a solid surface using lubrication theory [66,67]. They developed a single equation which considers both macroscopic evaporation and microscopic liquid adsorption. They concluded that both evaporation and thermocapillarity affect against droplet spreading and the rate of decay of contact angle due to evaporation is different than previous studies.

Whenever a droplet is being dried out, two effects are competing against each other, one tend to spread the droplet (capillarity), while the other induces a flow toward the acme of the droplet (Marangoni effect). The first one increases the evaporation rate whereas the second reduces it. Maki and Kumar were able to simulate both effects numerically using computational fluid dynamics (CFD) [7]. They employed the lubrication approximation to simplify the Navier-Stokes equations and solved the coupled system of equations by finite difference method using moving overset grid. They showed that Marangoni effect leads to skin formation in the droplets with colloidal suspensions. Due to an experimental study by Chao and Zhang [68], the evolution of contact diameter in an evaporating droplet can be divided into four stages: initial spreading, spreading-evaporation balance, evaporation-dominating contraction, and final rapid contraction. According to this report, thermocapillary convection shortens the second stage to become undetectable and its effect on contact angle depends on the evaporation rate.

Readers are referred to the other distinguished experimental attempts [69,70,71,72] and numerical models [73,74,75,76,77,78,79] on this subject for further information. The review papers by Erbil [80], Zhong *et al.* [81], and Kovalchuk *et al.* [82] also provide a complete collection of publications on the subject.

## 5. Thermocapillary-Induced Droplet/Bubble Actuation

### 5.1. Droplet Spreading

**Figure 5:**
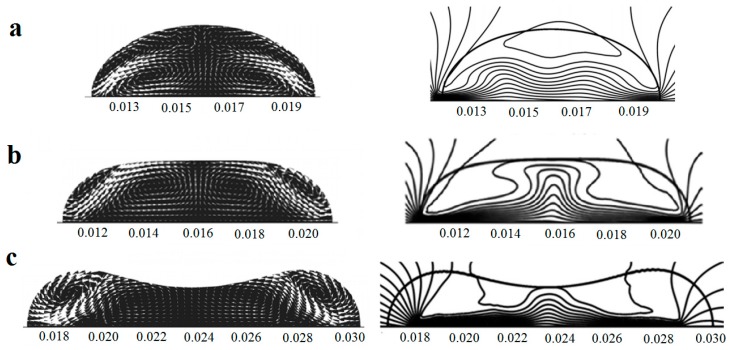
The flow field (left figures) and isotherms (right figures) inside the droplet with radius of *R* = 4 mm at *t* = 3.5 s (**a**), *R* = 5 mm at *t* = 3.5 s (**b**), and *R* = 5.5 mm at *t* = 2.5 s (**c**). Reprinted with permission from [86]. Copyright 2010, AIP Publishing LLC.

Droplet spreading on a heated solid substrate has been modeled numerically by several research groups. In 1999 Benintendi and Smith studied the effect of the slip coefficient and also the mobility capillary number on the spreading of a droplet on a heated/cooled solid substrate [83]. They compared their results with the works done by Ehrhard [84], and also Haley [85]. Thermocapillary-buoyancy convection of the liquid droplets on horizontal solid substrate subject to uniform temperature gradient has been numerically studied by Nguyen and Chen [86]. They used the finite element method to solve the Navier-Stokes equations and categorized the themocapillary-buoyancy migration/deformation of the droplets based on their size. Figure 5 shows the flow pattern and the isothermal contours of the droplets and the change of their response to temperature gradient with increasing the radius. According to this observance, increasing the droplet size and/or the temperature gradient can eventually lead to droplet splitting.

Krapetsas *et al.* extended it to the next level and studied the evolution of a droplet and changes of its contact angle in a non-isothermal inclined flat plate in 2013 [8]. They employed finite element method, and implicit Euler method for discretization in space, and time, respectively. In another numerical simulation, Karapetsas and his group investigated the droplet spreading behavior on a non-uniformly heated substrate for liquid droplets with non-monotonic surface tension-temperature dependence [87]. They assumed for the liquid droplets in case, the surface tension has a peak with respect to changing the temperature and they assumed that this peak is a minimum. The outcome was somehow similar to what Nguyen and his group observed which is shown in Figure 5. As a newer approach to the problem, Liu and coworkers developed a lattice Boltzmann algorithm to solve the flow field inside a droplet affected by thermocapillarity on the solid surface [88]. They succeeded to capture the flow field for different contact angles.

Certain conditions are necessary in order for the spread droplet to start moving on the solid surface due to surface tension gradient. Brzoska *et al.* studied the behavior of a droplet on nonwettable solid surfaces in reaction to a horizontal temperature gradient experimentally [89]. According to their findings a critical radius is defined for the droplets which is inversely proportional to the temperature gradient and independent of viscosity. Only the droplets whose radius is greater than the critical radius will move, and their velocity is linearly dependent on temperature gradient and inversely proportional to the dynamic viscosity of the droplet. Large droplets (with *R* > 6 mm) change their shape, due to gravity, from circular to two straight-line segment shape in the direction of the ∇T. Chen *et al.* studied the physics of droplet manipulation on a supported substrate experimentally and theoretically [90]. They calculated the threshold force for manipulation inception and the droplet velocity after depinning as a function of droplet size and composition, and thermal gradient. They followed the developed theory by Ford and Nadim [91]. Pratap and coworkers studied the thermocapillary motion of a droplet on a solid surface [92]. They studied the effect of droplet size and temperature gradient on droplet velocity both experimentally and theoretically. They also figured that the effect of droplet hysteresis is minimal and the resulting critical droplet size is independent of temperature gradient. This result is contradictory to what Brzoska reported. Baier *et al.* worked on the thermocapillary fluid motion on superhydrophobic surfaces theoretically [93]. They assumed the Cassie-Baxter state of flowing a thin film over an array of superhydrophobic pillars and developed a formula for migration velocity in the Stokes limit. They showed that due to the large contact surface to volume ratio of the liquid in this specific case relatively large velocities can be obtained.

**Figure 6:**
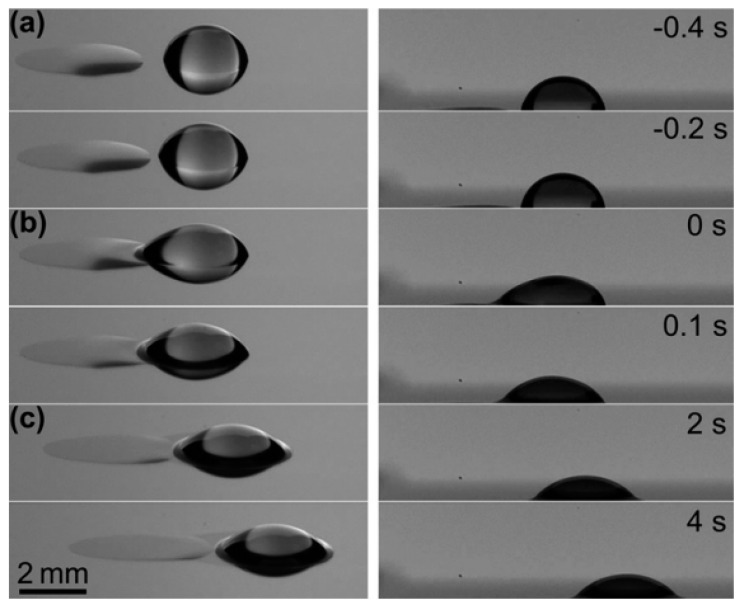
Thermocapillary actuation of a binary heptanol-water drop. Stage (**a**): heptanol spread against the temperature gradient; Stage (**b**): heptanol and water drop mixed together; Stage (**c**): the binary drop was driven to the cold side. Reprinted with permission from [94]. Copyright 2011, AIP Publishing LLC.

Droplet actuation on solid substrates is a challenge for the cases where the substrate is highly hydrophobic. Zhao *et al.* proposed a remedy for this problem and conducted experiments to enhance droplet actuation on hydrophobic substrates [94]. A fairly high hydrophobicity was produced by coating the silicon substrate with parylene for the purpose of the experiment. They showed that encapsulation of water droplets with long-chain alcohols such as heptanol can increase the contact angle of the binary drop (as is shown in Figure 6) and hence increase its drift velocity due to thermocapillary actuation.

### 5.2. Bubble/Droplet Migration

**Figure 7:**
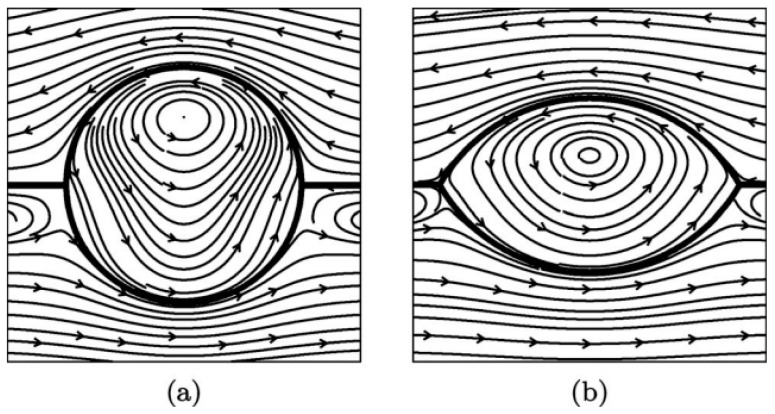
The droplet shape and streamlines of the flow in the *y* = 0 plane. The value of the surface tension ratio γ_12_ = 0 corresponds to a spherical droplet (**a**) and γ_12_ = 1 results in a slender droplet (**b**). All other parameters are fixed at unity. Reprinted with permission from [99]. Copyright 2009, AIP Publishing LLC.

The periodic fluid flow in and around a drop or a bubble eventually lead to its movement inside the carrier fluid. The motion of a confined liquid layer due to surface temperature gradient has been discussed by Pimputkar and Ostrach [95], and also by Sen and Davis [96] in two separate studies. As the result of this motion, droplets and bubbles have been reported to be actuated in such a bulk flow produced by thermal gradient either in a liquid [97,98], or at the interface of two liquids [99] as shown in Figure 7. An exact solution for the bubble/droplet velocity and its shape immersed in another liquid has been found by Balasubramaniam and Chai for small Marangoni numbers and any arbitrary Reynolds numbers [100].

In another phenomenon, “Bubble sweeping and circling”, the motion of the small bubbles around a larger one plays an important role in boiling heat transfer. Zhou *et al.* visualized this phenomenon during subcooled nucleate boiling of deionized water over a micro platinum wire [101]. They captured different types of bubble circling in their experiment, such as sweeping, departing, rotating, returning, and chasing of a group of small bubbles around a big one. It turned out that the thermocapillary force along with viscous force are major reasons in creating bubble sweeping which by itself affects the heat transfer rate especially at the microscale. The same group has published the numerical and theoretical study of the same phenomenon two years later [102].

It is more than two decades that researchers have also been focusing on solving the thermocapillary migration problems numerically. In 1996, Haj-Hariri *et al.* simulated the three dimensional thermocapillary motion of deformable drops under constant temperature gradient in a carrier liquid [103]. They observed that the shape change of the drops to either oblate or prolate spheroids retards the thermocapillary motion. Years later, Ajaev and Homsy solved the motion of a bubble inside a micro channel heated from the bottom numerically [104]. They used the lubrication theory for a long vapor bubble which accumulates most of the channel volume with the wall temperature around the bubble set to be constant. Mazouchi and Homsy analyzed the reverse flow of the liquid in thermocapillary migration of a confined bubble in a rectangular channel and they extended the results to cross sections of multiple sides and asymptotically to cylinders [105]. They assumed zero gravity in their theoretical study and proved by experiment that this assumption only holds in case of small vertical dimension of the channel [106]. Wang *et al.* [107], Yin and coworkers [108,109], and also Glockner and Naterer [110] used finite element, finite difference, and finite volume methods respectively along with the front-tracking scheme to simulate the thermocapillary migration of a single spherical non-deformable drop. Three years later, Chang *et al.* took the effect of droplet deformations into account in their finite difference simulation of thermocapillary migration [111]. They proved that increasing the temperature leads to increase in deformation rate due to reduction of the surface tension. They have also shown that change of the droplet velocity due to deformation is dependent on the relative density of the drop to the carrier fluid. A theoretical study has been performed on a steady non-isothermal two-phase flow by Choudhuri and Sekhar [112]. They calculated the thermally-induced drag and torque (thermocapillary drift) on the droplet. In another attempt Glockner and Naterer developed a theoretical and numerical formulation to analyze the thermocapillary force imposed on a confined advancing droplet during its revolution to migrate [113]. They showed that the thermocapillary force experienced by the receding contact line of the droplet increases until it starts moving and after that it starts decaying. Yang *et al.* from the same group also worked on theoretical formulation of contact angle changes and motion of a confined droplet in a microchannel under temperature gradient [114]. Recently Lai *et al.* also simulated the confined drop motion due to thermocapillary using the volume-of-fluid method [115]. In a more recent study Thermocapillary motion of drops was simulated by Liu and coworkers using lattice Boltzmann method [116,117]. They captured the fluid flow in and around a droplet along with the temperature contours for different Marangoni numbers. Another theoretical study has been done by Baird and Mohseni on modeling velocity of discrete microdroplets [118]. Separate discussion and modeling for electrowetting on dielectric (EWOD), dielectrophoresis, continuous electrowetting (CEW), as well as thermocapillary pumping is given.

#### 5.2.1. Mechanical Heating

The source of local temperature gradient might be in contact or remote from the experimental platform. A simple way of producing heat to the sample is by embedding metal heaters. Hadland *et al.* performed experiments to capture thermocapillary migration of air bubbles and fluorinert drops in silicone oil under reduced gravity [119]. Their ultimate purpose was to measure the migration velocity of fluorinert oil drops and air bubbles immersed in the silicone oil as a function of different factors such as the distance from the wall, and Marangoni and Weber numbers. Minimization of gravitational and buoyancy effects on the platform, would enforce limitations on vertical temperature gradients as well as the size of the bubbles and drops. They covered a wide range of Reynolds and Marangoni numbers and compared their data with the earlier experimental and numerical results. It turned out that the deformation of the bubbles and droplets under their experiments conditions was negligible. They also studied the effects of Marangoni and Weber numbers on the velocity of the bubbles and droplets. Dependency of density and viscosity on temperature and its effect on transient and steady motion of fluorinert oil and air bubbles in silicone oil was also discussed in their report. In another experimental attempt Treuner *et al.* observed bubble migration in paraffin liquids under reduced gravity for high Reynolds and Marangoni number of up to *Ma* = 2500 [120]. They reported that high Marangoni number condition imposes a slight but important correction to the previous results. Their framework was completed by including a theoretical study of the subject the results of which were in agreement with previous studies. Years later a similar experiment was also conducted by Xie *et al.* for Marangoni numbers of up to 5500 [121].

Young *et al.* designed another experiment to correlate the bubble size and the vertical temperature gradient with the velocity of the bubble in a vertical cylindrical chamber [122]. They have found a critical bubble size at which the bubble can stay stationary under a fixed vertical temperature gradient. Thermocapillary-induced migration of drops has been theoretically formulated by Zhang *et al.* considering the inertia and gravitational effects [123]. Harper *et al.* showed that the temperature-induced surface tension gradient around a bubble confined in a liquid bath is not big enough to induce actuation unless surface active substances are present [124]. In another study, thermocapillary was compared against solutocapillary as thermal and chemical factors in bubble motion confined in liquid bath [125].

**Figure 8:**
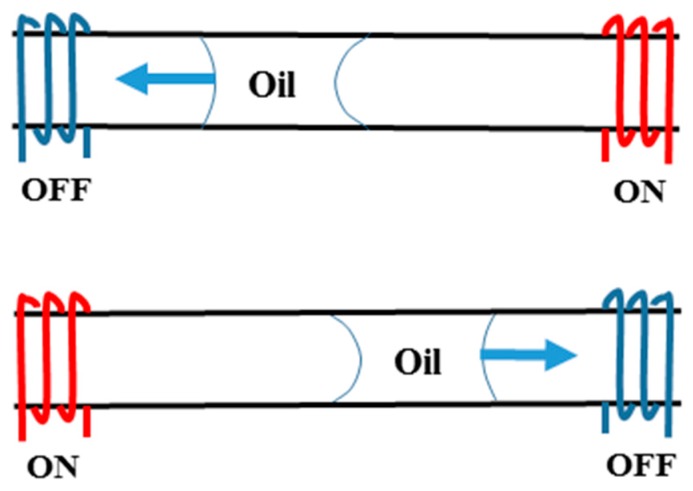
Periodic back and forth movement of the oil plug due to changing the direction of thermocapillary force by switching the left and right heaters.

Movement of a silicone oil plug in a channel using thermocapillary effect was observed and simulated using three coil heaters by Nguyen’s group [126,127,128] (as is shown schematically in Figure 8) in order to realize the polymerase chain reaction (PCR) of deoxyribonucleic acid (DNA). This observance was also formulated theoretically by the same group [129,130] and proved that this motion becomes chaotic by increasing the frequency [131].

**Figure 9:**
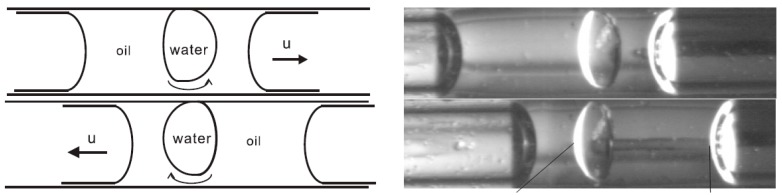
Encapsulation of a water drop inside an oil plug in a microtube under thermocapillary effect observed by Jiao *et al.* Reproduced with permission from [132], Z. Jiao, N.-T. Nguyen, and X. Huang, “Thermocapillary actuation of a water droplet encapsulated in an oil plug”, *Journal of Micromechanics and Microengineering,* vol. 17, p. 1843, 2007. Copyright 2007, IOP Publishing, all rights reserved.

They also succeeded to encapsulate a water drop inside an oil plug under some certain conditions and transport the aqueous unit under cover of oil as is shown in Figure 9 [132]. Readers are referred to [133] and [134] for more details about their work.

#### 5.2.2. Optical Heating

**Figure 10:**
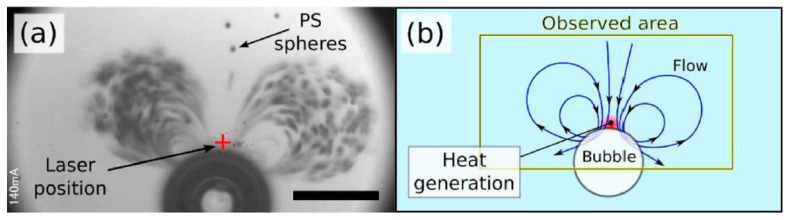
(**a**) Microscopic image of the rapid flow around the micro bubble induced by the photothermal conversion. The cross mark shows the laser position, the small black dots are the polystyrene (PS) spheres, and the big black circle is the micro bubble with the diameter of around 65 µm. Scale bar: 50 µm. (**b**) Sketch of the flow around the bubble. Reprinted with permission from [135]. Copyright 2015, AIP Publishing LLC.

Another way of supplying the heat which is more convenient and needs almost no fabrication cost is heating through emission of a focused laser beam. Namura *et al.* demonstrated the local Marangoni flows around micro bubbles induced by a localized heating source with a laser beam [135]. They radiated a laser beam on Au/Ag substrate and reported strong main flow toward the bubble along with circulations around it, as is shown in Figure 10. The strength and exact shape of the local flows changes with the position of the beam irradiation. The purpose of this experiment is to control the temperature gradient of the bubble, as well as sorting the microparticles such as polystyrene.

**Figure 11:**
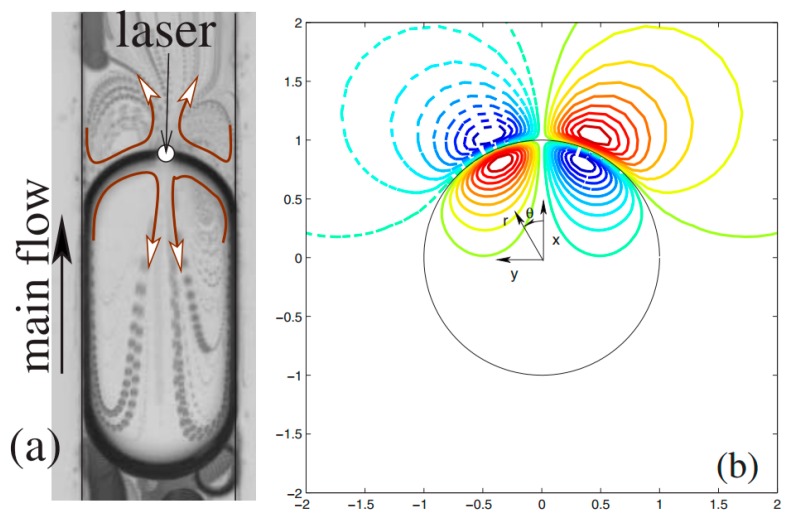
(**a**) Experimental, and (**b**) numerical results of the fluid flow that forms around a laser spot on the interface of the droplet stopping it from further movement due to thermocapillary phenomenon. Reprinted figures with permission from [136], C.N. Baroud, J.-P. Delville, F. Gallaire, and R. Wunenburger, *Physical Review E,* vol. 75, p. 046302, 2007. Copyright 2007, American Physical Society.

**Figure 12:**
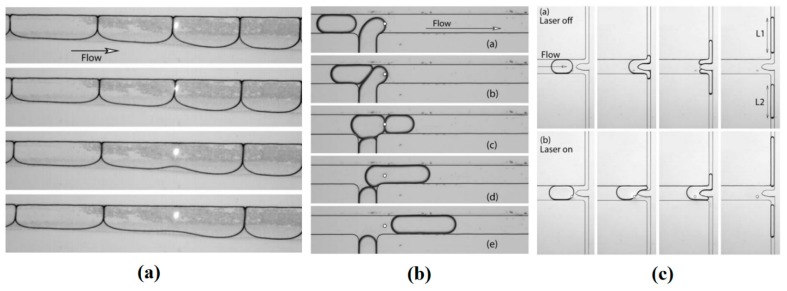
(**a**) Localized fusion and merging of the two adjacent drops; (**b**) Blocking the first drop by a laser valve until the second drop arrives and merging the two by another laser beam at the interface; (**c**) A droplet sampler that decides precisely what portion of the drop should go to each specific outlet. Republished with permission of Royal Society of Chemistry, from [140], “An optical toolbox for total control of droplet microfluidics”, C.N. Baroud, M.R. de Saint Vincent, and J.-P. Delville, *Lab on a Chip,* vol. 7, pp. 1029–1033, 2007; Permission conveyed through Copyright Clearance Center, Inc.

Baroud and coworkers worked on the concept of producing microdroplet valve by using a focused laser beam spot in an enclosed droplet generation system [136]. They found out that Marangoni circulations induced around the laser spot (as shown in Figure 11) can stop the droplet from traveling forward and hence can be interpreted as a hand-free, fabrication-free microdroplet valve. They formulated this phenomenon and proposed a scaling law for the net force submitting on the droplet [137]. Their finding is consistent with Glockner and Naterer’s numerical result on studying flow field [138] and heat transfer [139] of a confined droplet under thermocapillary effect. They studied the reason for this phenomenon by analyzing the force balance on the droplet. This behavior was also applied for droplet formation, transport, division, and fusion (Figure 12) [140].

**Figure 13:**
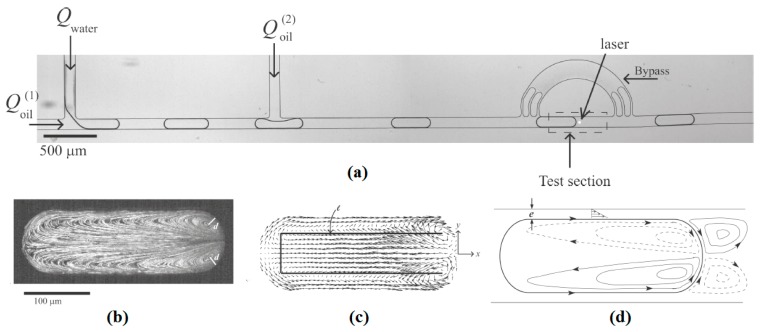
Studying the flow pattern inside a droplet by emitting a point laser beam on its interface. (**a**) The experimental apparatus, (**b**) the actual flow pattern inside a confined drop due to two point laser beams located on its interface symmetrically, (**c**,**d**) simulated flow pattern under the same conditions as in (**b**). Reprinted with permission from [141]. Copyright 2009, American Chemical Society.

This phenomenon was shown later by Verneuil *et al.* from the same group [141]. They designed an experiment and captured flow field inside a confined droplet exposed by two laser beams (Figure 13). They also measured the value of the force imposed to the droplet by a micro-dynanometer, correlated it to the intensity of the laser beams, and derived the maximum force that a droplet stopped by a specific laser beam can tolerate before it starts moving.

Ohta *et al.* also demonstrated the bubble manipulation in silicone oil using optical heating [142]. Since they supplied the heat remotely to the silicone substrate, their method was successful in manipulating bubbles in transparent liquids. G. Faris along with his group also performed a series of experiments and succeeded to manipulate water droplets immersed in decanol by focusing a laser beam on them and producing Marangoni convection [143,144,145]. Droplets in a wide range of volume from 10 to 1000 pL, with and without surfactant are tested and velocities in the order of few mm/s and rate of mixture of less than a minute were reported by them. Further review on optofluidics and optothermal manipulation is available in references [146,147,148,149].

### 5.3. Thermocapillary Mixing

**Figure 14:**
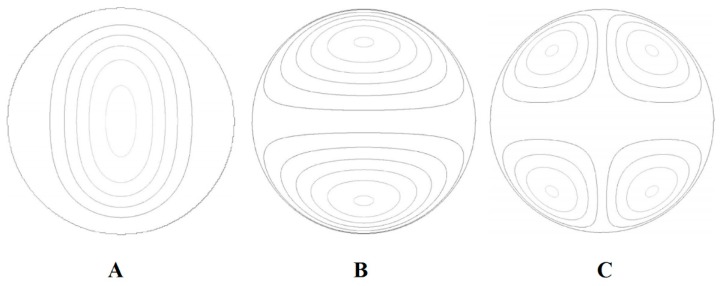
Streamlines of the Taylor (**A**), dipole (**B**), and quadrupole (**C**) flow at the midplane of the droplet. Reprinted with permission from [150]. Copyright 2005, AIP Publishing LLC.

**Figure 15:**
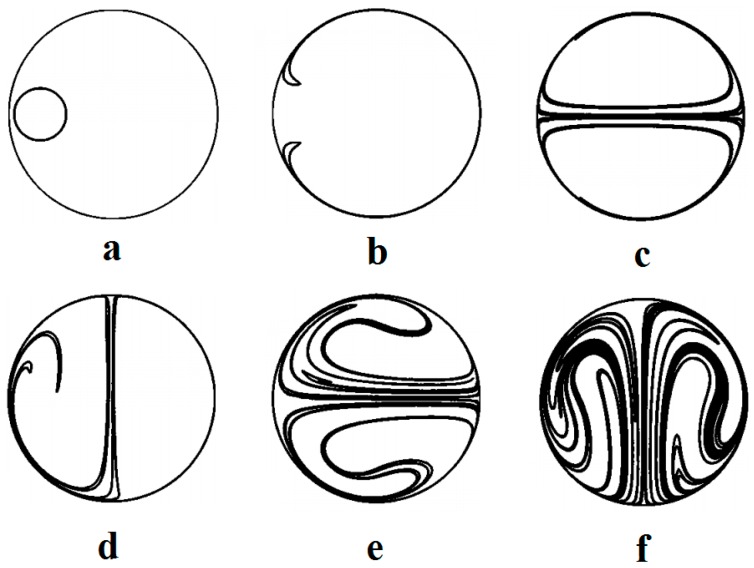
Advection of dye by the dipole flow shown in the midplane of the droplet. The initial state (**a**) and stretching in steady dipole flow at *t* = 6 (**b**) and *t* = 24 (**c**). Stretching and folding in a time-periodic flow obtained by rotating its direction by 90° in the horizontal plane every six time units. At *t* = 12 (**d**), *t* = 18 (**e**), and *t* = 24 (**f**). Reprinted with permission from [150]. Copyright 2005, AIP Publishing LLC.

During the thermocapillary actuation Marangoni effect can be seen even in the smallest scales of the flow and within the bubbles and droplets. As one of these effects, the nature of thermocapillary-induced chaotic mixing in the droplets has been studied numerically by Grigoriev [150] in continuation of his analytical work on droplet chaotic mixing [151]. It is shown that, for complete mixing with chaotic advection in a microdroplet, all the invariant surfaces serving as barriers for transport are deformed (Figure 14). For this purpose, experiments have been performed on the microdroplet with dye using temperature gradient, and the results are shown in Figure 15. Vainchtein *et al.* used these results and expanded a theoretical basis for this advection chaotic mixing [152]. They claimed that two different metrics are involved with the mixing quality. The first one calculates the volume fraction of chaotic *versus* regular streamlines while the second one is involved with the time required for homogenization of chaotic mixing inside the droplet.

**Figure 16:**
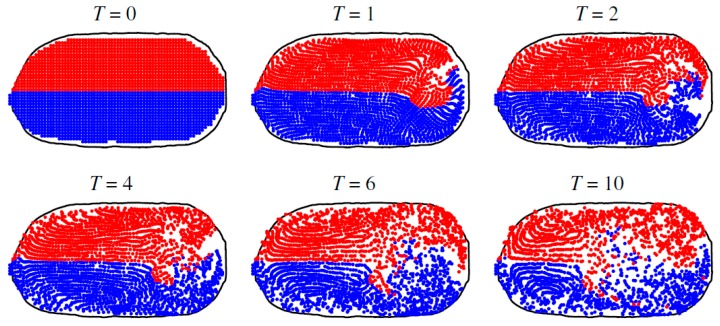
Snapshots of the position of passive tracer showing the enhancement of mixing of the lower and upper halves of the droplet using two stationary (not altering) laser beams. Reproduced with permission from [155], M.L. Cordero, H.O. Rolfsnes, D.R. Burnham, P.A. Campbell, D. McGloin, and C.N. Baroud, “Mixing via thermocapillary generation of flow patterns inside a microfluidic drop”, *New Journal of Physics,* vol. 11, p. 075033, 2009. Copyright 2009, IOP Publishing & Deutsche Physikalische Gesellschaft. CC BY-NC-SA.

Due to the small length scale in microfluidics, the turbulence contribution in mixing is ruled out and this makes mixing much harder than that in large scales. The theory of continuous mode free surface thermocapillary mixing of thin liquid rivulets and the relevant time and length scales was discussed by Darhuber and coworkers [153]. According to their work, for large surface to volume ratios, three different mixing regimes exist, namely: purely diffusive dynamics, Rhines-Young sheer augmented diffusion, and Taylor-Aris Dispersion. A method proposed by Muruganathan *et al.* was to intentionally create substrate surfaces with microcracks (microfolds) to induce vortical flow with finite vorticity in presence of thermocapillary to enhance mixing performance [154]. As another method to enhance mixing in microscale, Cordero *et al.* in the continuation of their work on using laser beam in manipulation of confined droplets, proposed that if the two laser beams emitted on the interface of the droplet have the switching frequency less than response rate of fluid, the symmetry in fluid pattern inside the droplet will vanish and this asymmetry increases the mixing rate (Figure 16) [155].

### 5.4. Thermocapillary Coalescence and Nonwetting

In the process of thermocapillary motion, bubbles and droplets might break down and rejoin to one another due to short-range forces and instabilities. Marangoni convection in and around the drops in presence of thermocapillarity, the temperature difference between the droplet(s) and the surrounding fluid or the solid substrate develops a draining film between the two droplets or the droplet and the substrate. This lead to a phenomenon named noncoalescence or nonwetting in which droplets do not completely coalesce after collision.

In 1998 Monti *et al.* studied the behavior of a pendant droplet between two solid surfaces both experimentally and numerically [156]. They reported that if the substrate being heated and/or the lower substrate being cooled there will be some specific conditions under which the droplet does not wet the lower substrate and deforms instead. The reason for this counterintuitive phenomenon is reported to be the pressure balance between the droplet and the air film which exists under the droplet due to the Marangoni effect. Khan *et al.* also showed that formation of noncoalescent drops (NCDs) on the solid/air interface is dependent on the Weber number [157]. For the case of ink jet printing, critical weber number of 130 was reported over which generation of NCDs became possible. Similar to Monti’s report, existence of air cushion between the drop and solid surface with greater thickness than the van der Waals range of action was reported as the reason for NCDs production. The behavior of hot droplets in proximity of a cold wall is also observed numerically and the nonwetting phenomenon is reported by Chen and coworkers [158]. The temperature difference and the walls distance has contributions to this behavior. It was shown that the phenomenon does not happen if the droplet is colder than the wall.

Two colliding droplets might end up coalescing depending on different factors such as collision speed, angle, droplets composition, as well as their temperature. Choi and Lee published their work in 2013 on numerical simulation of film drainage between two coalescing droplets [159]. They captured the phenomenon in wide range of relative interfacial tension and categorized it as fast, intermediate, and delayed drainage. They also realized that there is a secondary flow developing in the carrier fluid due to the film drainage for the cases where the two droplets are immiscible. Savino and Monti—in another attempt—simulated the behavior of two non-coalescing droplets of the same fluid pressed against each other in presence of thermocapillary convection, numerically [160]. They used fourth order Runge-Kutta method and solved Gauss-Laplace hydrostatic equation to assess the droplet deformation, pressure distribution, and air film thickness between the two droplets. The flow field inside the droplets under thermocapillary convection is also captured. Their numerical results were in acceptable qualitative agreement with experimental data.

**Figure 17:**
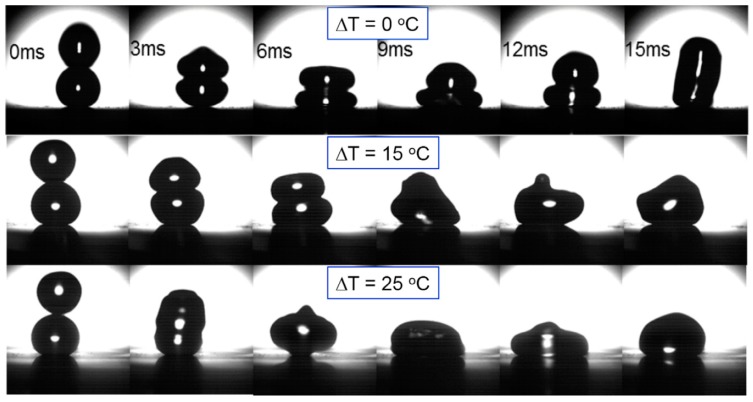
Head-on collision of binary droplets with increased temperature of the incoming droplet at releasing height of 13 mm (*T*_stationary_ = *T*_room_ = 25 °C, Δ*T* = *T*_incoming_ − *T*_stationary_). Reprinted by permission from Macmillan Publishers Ltd: Scientific Reports [162], Copyright 2014.

The effect of relative surface tension on coalescence of droplets of immiscible liquids has been studied by Blanchette [161]. Yi and coworkers also studied the effect of droplets temperature on the probability of coalescence on superhydrophobic surfaces experimentally [162]. The superhydrophobic surface was fabricated by silver-assisted etching of the silicon substrate and silanization with fluorosilane. Different steps of collision were captured by high-speed camera for different temperate differences as shown in Figure 17.

**Figure 18:**
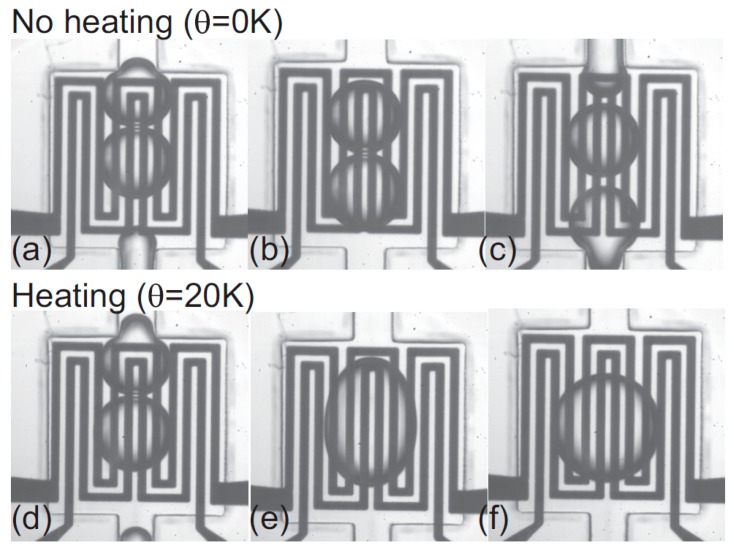
Using a heater to study the coalescence of two droplets under thermocapillary effect. Reprinted with permission from [163]. Copyright 2012, AIP Publishing LLC.

Nguyen’s Group conducted an experiment on thermocoalescence of two immiscible droplets [163]. They reported that the two droplets on top of a heater slow down as they approach each other and they coalesce only by heating more than a critical value under which no coalescence take place (as is shown in Figure 18). Noncoalescence was also observed in microgravity condition by Dell’Aversana *et al.* [164].

**Figure 19:**
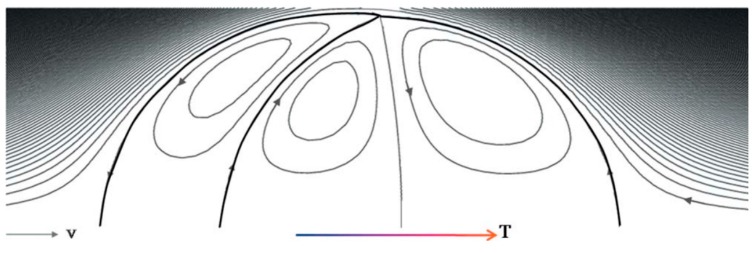
Streamline pattern of a compound drop moving with temperature gradient. Reproduced with permission of Cambridge University Press, from [168], L. Rosenfeld, O. Lavrenteva, and A. Nir, “On the thermocapillary motion of partially engulfed compound drops”, *Journal of Fluid Mechanics,* vol. 626, pp. 263–289, 2009; Permission conveyed through Copyright Clearance Center, Inc.

The behavior between two coalescing bubbles or droplets within a continuous phase has also attracted attention due to the implications of this phenomenon for various applications. Wang and Davis solved the population dynamics equations numerically in order to analyze the phase separation and droplet size distribution in a nonhomogeneous dispersion [165]. Buoyancy force lead the droplets to settle and coalesce, whose rate is reported to increase at the beginning and decrease after a while. The behavior of the drop cloud in thermocapillary motion has been studied numerically by Nas *et al.* for both mono-dispersed and poly-dispersed cases [166]. They used front tracking/finite difference method and realized that layers of drops form perpendicular to temperature gradient for moderate Reynolds and Marangoni numbers. Droplet engulfment or encapsulation phenomenon has been a subject of investigation and application. Lavrenteva *et al.* published the results of their theoretical studies on partial engulfment of slightly deformable (small Capillary number) compound droplets in an immiscible non-isothermal carrier fluid [167,168]. They showed that the partially engulfed droplets consist of three spherical surface segments which are subject to significant change due to droplet propagation in non-isothermal case (Figure 19). These changes might lead to separation or full engulfment. They also simulated the motion of a compound drop due to Marangoni effect which can be unpredictably against the temperature gradient under specific conditions [169].

**Figure 20:**
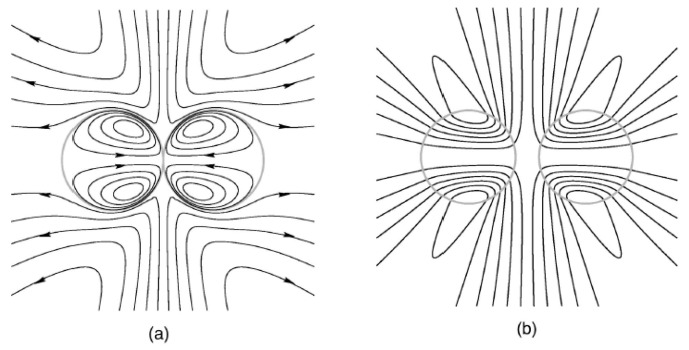
Streamlines of spontaneous thermocapillary flow in and around two spherical drops in contact (**a**), and at a separation distance (**b**). Reprinted with permission from [171]. Copyright 2002, AIP Publishing LLC.

Satrape studied the theory of coalescence of two undeformable bubbles in thermocapillary motion in the creeping flow regime and very small Peclet number [170]. He also proposed a statistical model based on discrete stochastic collection equation to model coalescence in a cloud of bubbles in terms of size distribution, volume fraction, average temperature gradient and other characteristics of the system. The axisymmetric interaction of two viscous drops under thermocapillary forces was studied by Berejnov *et al.* in 2002 [171]. The Marangoni flow streamlines are shown in Figure 20.

**Figure 21:**
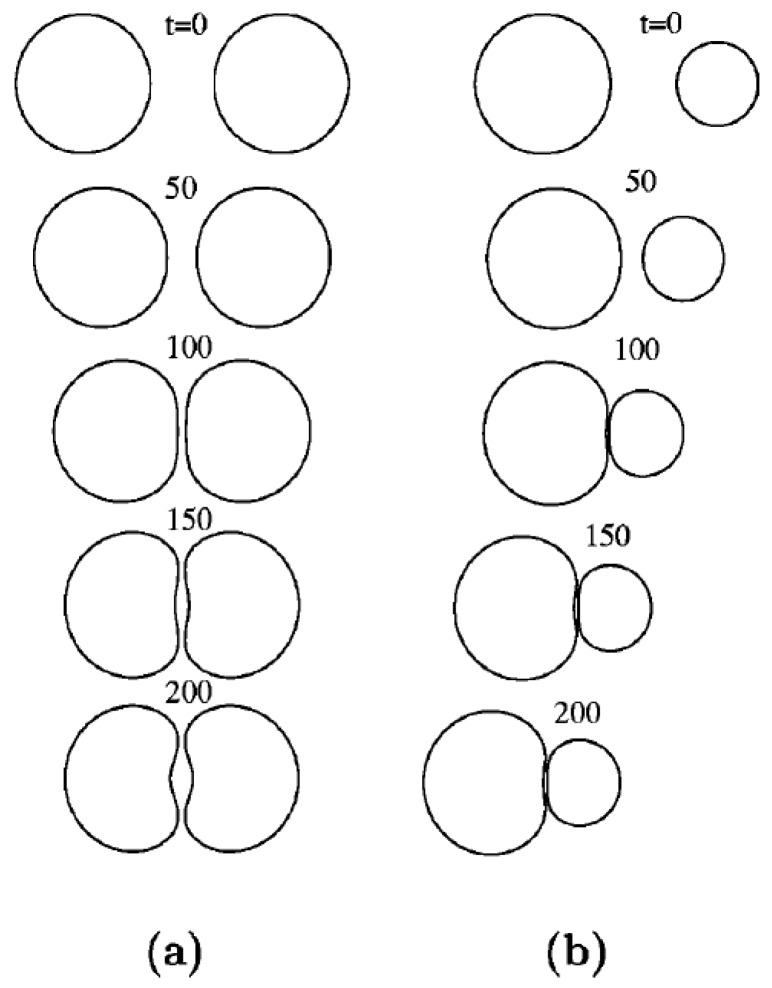
A display of a dynamic deformation and migration of two interacting drops. (**a**) *R* = 1 *Ca* = 1; (**b**) *R* = 0.6 *Ca* = 0.67 (with *R* being the ratio of the radius of two drops and *Ca* the capillary number). Reprinted with permission from [171]. Copyright 2002, AIP Publishing LLC.

They performed simulation for two droplets of the same size as well as two of different sizes. For the droplets of equal size it turned out that for high capillary numbers the normal forces exerted by the liquid film between the two droplets equates with the Marangoni force pushing the droplets toward each other. This will lead to the deformation of both droplets (Figure 21a). Whereas capillary number approaches zero the Marangoni stresses become dominant and the liquid film between the droplets diminishes eventually. If one droplet is smaller than the other one it becomes more resistant toward deformation therefore the deformation rate of the larger droplet is more (Figure 21b).

Therefore, coalescence of two droplets can be defined as unification of the boundaries of the two drops after collision. Certain conditions exist under which collision of a droplet to either a solid/liquid substrate or a second drop does not lead to coalescence. This phenomenon so called noncoalescence or nonwetting is caused by Marangoni convection followed by developing of a draining fluid film between the two boundaries in cases where thermocapillarity exists. More detailed discussion on noncoalescence is provided by Neitzel and Dell’Aversana in form of a review on noncoalescence and nonwetting behavior of liquids [172].

### 5.5. Drop Manipulation on Free Surface

**Figure 22:**
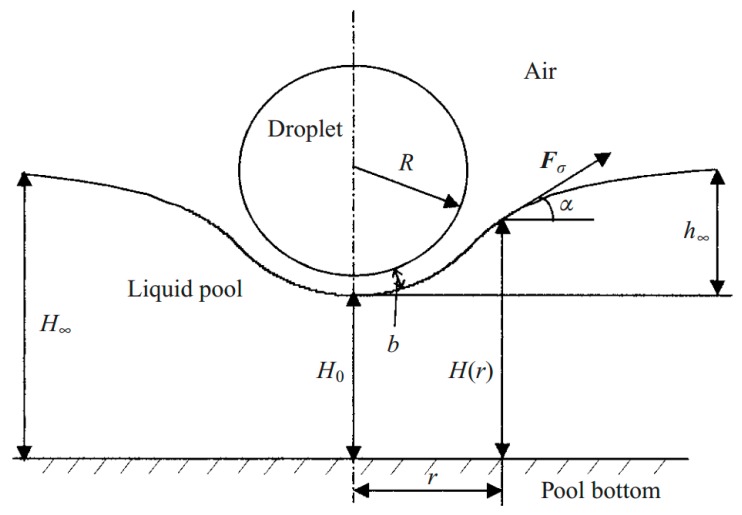
Liquid surface deformation in existence of thermocapillarity and levitation of a spherical droplet with an air film separating it from the liquid film. Reproduced with permission of Cambridge University Press, from [175], R. Savino, D. Paterna, and M. Lappa, “Marangoni flotation of liquid droplets”, *Journal of Fluid Mechanics,* vol. 479, pp. 307–326, 2003; Permission conveyed through Copyright Clearance Center, Inc.

Taking advantage of noncoalescence phenomenon of a droplet on a liquid substrate, researchers focused on manipulating droplets on the liquid free surface using thermocapillarity. In 1988, Brochard worked on the equilibrium shape of the droplet on top of a liquid layer under the limit of low surface tension gradient [173]. He reported that for droplets under the Laplace length the equilibrium shape is spherical, while the droplets larger than this size turn to a pancake shape. He considered both chemical and thermal factors in producing surface tension gradient and observed a dual behavior from the droplet. Later, Sreenivas and coworkers showed that droplets can levitate on top of a liquid film under certain conditions [174]. They produced a radial flow along with a hydraulic jump on the liquid surface flow and observed that these two effects lead the droplets of certain size not to immerse. Following these studies, years later the phenomenon of the floatation of liquid droplets on the pool surface with different temperature has been discovered and reported by Savino *et al.* [175]. Presence of a circulating air film between the droplet and the liquid surface has been maintained by producing thermal Marangoni motion, which is proven both experimentally and theoretically in this paper (Figure 22).

**Figure 23:**
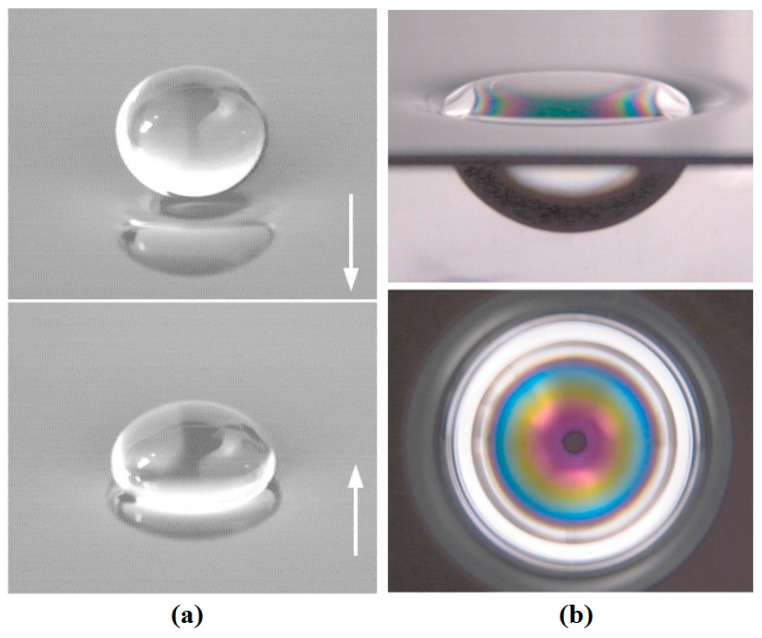
(**a**) Two photographs showing a drop of radius 2 mm as it bounces on the liquid surface. The arrows show the direction of bath motion. (**b**) A large floating drop as seen from the side (top). The interference fringes of the air film as observed when the drop is lit from the top with white light. The black dot at the center is the reflection of the camera’s lens. Reprinted figures with permission from [176], Y. Couder, E. Fort, C.-H. Gautier, and A. Boudaoud, *Physical Review Letters,* vol. 94, p. 177801, 2005. Copyright 2005, American Physical Society.

Two years later the same phenomenon was observed by Couder and coworkers with drops on top of the film of the same liquid [176]. They observed levitated drops on top of a vertically vibrating liquid film as is shown in Figure 23. It was seen that if the liquid film has vertical fluctuation small drops can be produced on top of the film and they can eventually grow up to millimeters of diameter. Even when the liquid becomes quiescent the drops can stay on top for a while, owing to the slowly draining air film (every 30 min) that was produced between the drop and the liquid film. They reasoned their observance with lubrication theory analysis.

**Figure 24:**
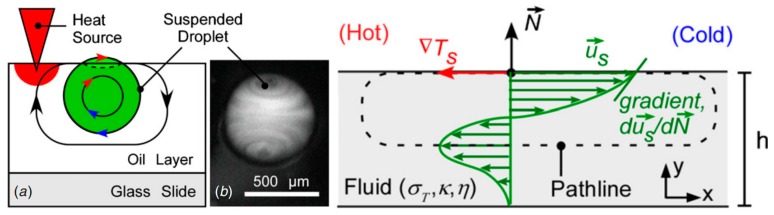
(**a**) Flow pattern in and out of a droplet in presence of thermal gradient, (**b**) flow pattern in a liquid film driven by surface temperature gradient. Reproduced with permission from [178], A.S. Basu and Y.B. Gianchandani, “Virtual microfluidic traps, filters, channels and pumps using Marangoni flows”, *Journal of Micromechanics and Microengineering,* vol. 18, p. 115031, 2008. Copyright 2008, IOP Publishing, all rights reserved.

A clear illustration of Marangoni convection inside the levitated drop on a free surface of a liquid bath which leads to translational motion of the drop was reported by Rybalko *et al.* in 2004 [177]. They produced the fluid flow inside the droplet by a focused laser beam and showed that changing the angle of the beam and its exact spot on the droplet leads to changing the translational motion direction of the droplet by 180°. This is one of the first efforts toward thermocapillary lateral manipulation of levitated droplets on liquids free surfaces. After that, Basu and Gianchandani published their work in 2008 in purpose of proposing a thermocapillary open surface microdevice with remote heaters [178]. They provided a detailed description on the flow pattern in both liquid film and levitated droplets in presence of local thermal gradient [179] as is shown in Figure 24. Their proposed device is discussed in “device applications” section of this review.

**Figure 25:**
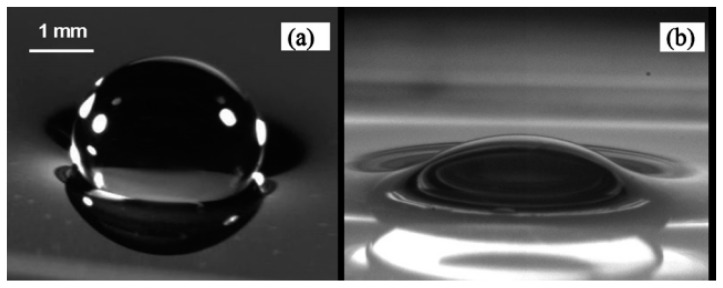
Aqueous drops resting on the free surface of fluorocarbon liquid (FC-43) in a spherical mode (**a**) and lens mode (**b**). Reprinted with permission from [181]. Copyright 2010, AIP Publishing LLC.

In pursuit of studying the liquid-liquid interaction with temperature change, in 2009 our group conducted a series of experiments by which it was proven that a droplet on top of an immiscible carrier liquid has a dual behavior toward temperature gradient based on its configuration (Figure 25) [180]. We also studied the dominant forces affecting this motion in different configurations and measured the velocity of the droplet at different distances from the heater.

**Figure 26:**
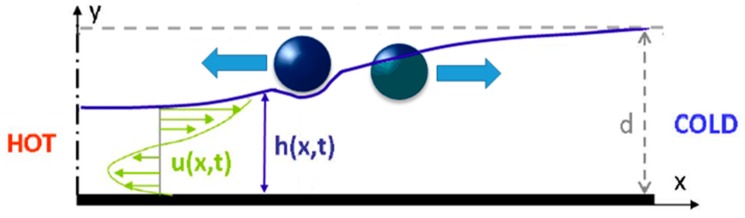
Deformation and flow generated at the free surface of thin immiscible liquid layer drives levitated-spherical droplets toward the hotter end of a thermal gradient. Copyright 2011, IEEE. Reprinted and modified, with permission, from [182], E. Yakhshi-Tafti, R. Kumar, and H. Cho, “Thermally-actuated high speed droplet manipulation platform”, in Proceesings of 2011 16th International Solid-State Sensors, Actuators and Microsystems Conference (TRANSDUCERS), 2011, pp. 1484–1487.

The physics behind this complicated dual behavior was reported [181]. We showed that formation of the Marangoni flow and hence the surface depression in the carrier liquid due to the imposed temperature gradient (as is shown in Figure 26) is the reason of the droplets moving toward/away from the heat source.

**Figure 27:**
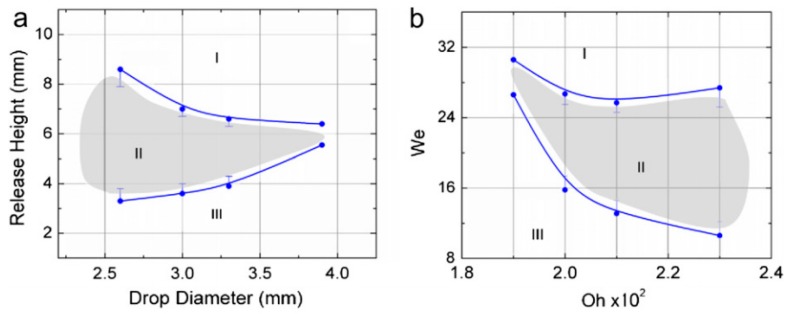
(**a**) Release height as a function of drop diameter, and (**b**) We as a function of Oh. (**I**) region in which drops collapse into submerged form; (**II**) region in which drops assume a spherical ball-shape above the surface; and (**III**) Region in which the drops bridge the gap between the dispensing tip and the liquid interface where free falling pendant drops cannot be formed. Reprinted from *Journal of Colloid and Interface Science,* vol. 350, E. Yakhshi-Tafti, H.J. Cho, and R. Kumar, “Impact of drops on the surface of immiscible liquids”, pp. 373–376 [183]. Copyright 2010, with permission from Elsevier.

At the same time, in 2010, our group published the results of the experiments on the effort for producing circular (levitated) droplets [183]. According to the results there is a range of release height and droplet size in relation to each other that can guarantee the production of levitated droplets. This important outcome was shown as a feasible area in height-diameter, and Weber-Ohnesorge plots (Figure 27).

**Figure 28:**
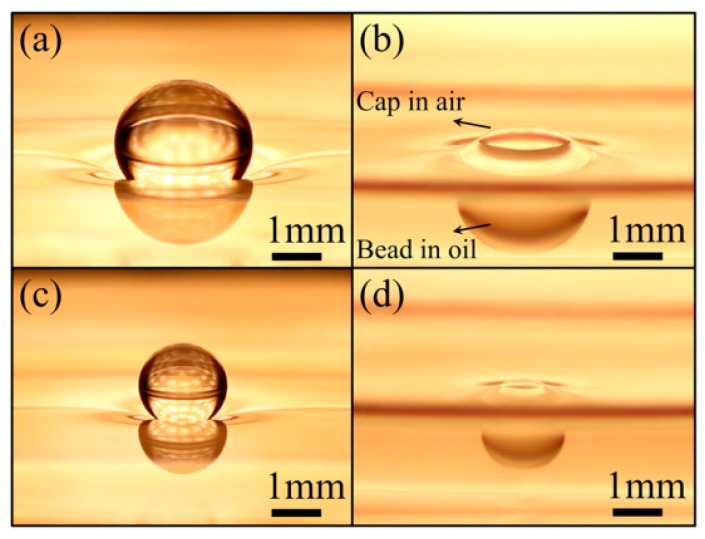
Stable configurations of aqueous droplets at oil-air interface. (**a**) Non-coalescent droplet resting on a stretched and deformed free surface. (**b**) Cap-bead droplet with triple contact line. (**c**,**d**) The effect of droplet size on the deformation of free surfaces. Reprinted with permission from [184]. Copyright 2013, AIP Publishing LLC.

These results were backed up by Shabani *et al.* in 2013 through a theoretical force and energy analysis [184]. Seeking the equilibrium configurations of a droplet on an immiscible liquid, we proved that it is in its minimum energy state either in levitated or submerged configuration (Figure 28). Further details about this work are available in references [185,186].

## 6. Device Applications

### 6.1. Thermocapillary Pumps, Mixers, and Actuators

**Figure 29:**
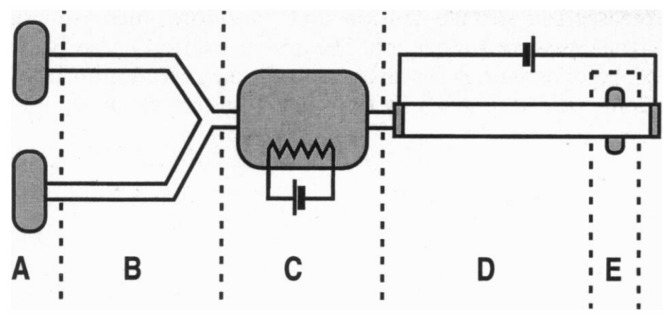
Schematic of an integrated DNA analysis device proposed by Burns *et al.* which had five different sections: (A) Injection entry ports; (B) thermocapillary pumping channels; (C) thermally controlled reaction chamber; (D) electrophoresis channel; and (E) DNA band migration detector. Reprinted with permission from [187], M.A. Burns, *et al.*, “Microfabricated structures for integrated DNA analysis“, *Proceedings of the National Academy of Sciences,* vol. 93, pp. 5556–5561. Copyright 1996, National Academy of Sciences, USA.

In 1996, Burns and coworkers proposed a multicomponent integrated DNA analysis device which included injection entry ports, liquid pumping channels, thermally controlled reaction chamber, electrophoresis channel, and DNA band migration detector [187]. The schematic of this device with all the components is shown in Figure 29.

**Figure 30:**
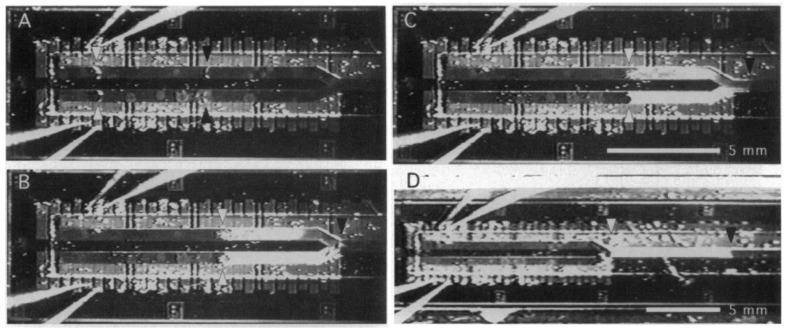
Droplet actuation and mixing in the Y-channel by thermocapillary effect. Reprinted with permission from [187], M.A. Burns, *et al.*, “Microfabricated structures for integrated DNA analysis”, *Proceedings of the National Academy of Sciences,* vol. 93, pp. 5556–5561. Copyright 1996, National Academy of Sciences, USA.

The second component in their device, which was responsible for delivering the drops to the reaction chamber, worked based on the thermocapillary concept. By switching the heater embedded beneath the channels sequentially, the droplets were moving from the injection unit toward the reaction chamber as shown in Figure 30. The transporting unit was made of bonding the glass channels to the silicon substrate. Their device is considered as one of the first thermocapillary based pumps.

**Figure 31:**
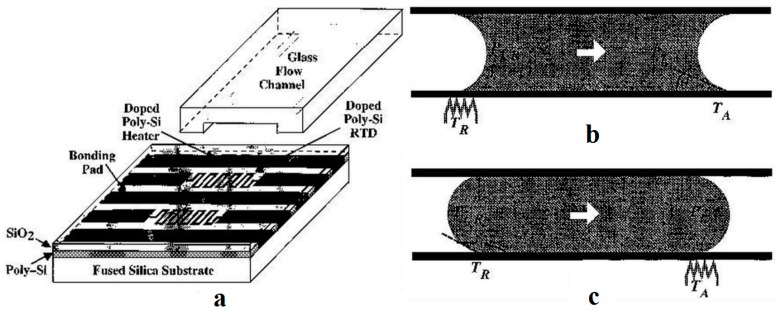
The thermocapillary pumping device proposed by Sammarco and Burns (**a**) actuating a confined droplet in a hydrophilic (**b**) and hydrophobic (**c**) channel. Reprinted with permission from [188], T.S. Sammarco and M.A. Burns, “Thermocapillary pumping of discrete drops in microfabricated analysis devices”, *AIChE Journal,* vol. 45, pp. 350–366. Copyright 1999, John Wiley and Sons.

Three years later, thermocapillary pumping (TCP) was described using a proposed microchannel device by Sammarco and Burns [188]. Their method is to heat one end of the confined droplet inside a microchannel to produce surface tension gradient in the droplet that initiates the movement. This method has been proven to be compatible with both hydrophilic and hydrophobic channel surfaces as shown in Figure 31. The advantage of this work compared to the previous proposed devices is that they have addressed the contact angle hysteresis of the drops (as the main drawback of their method) by using surfactants, converging channels, or external pressure.

Around the same time period, Takahashi and coworkers proposed a micro-oscillator based on periodic actuation of a bubble using thermocapillary effect [189]. Using the same approach, they fabricated two heaters and took advantage of both thermocapillarity and boiling heat transfer to produce a repeatable back and forth motion in a microbubble which could role as an oscillator.

**Figure 32:**
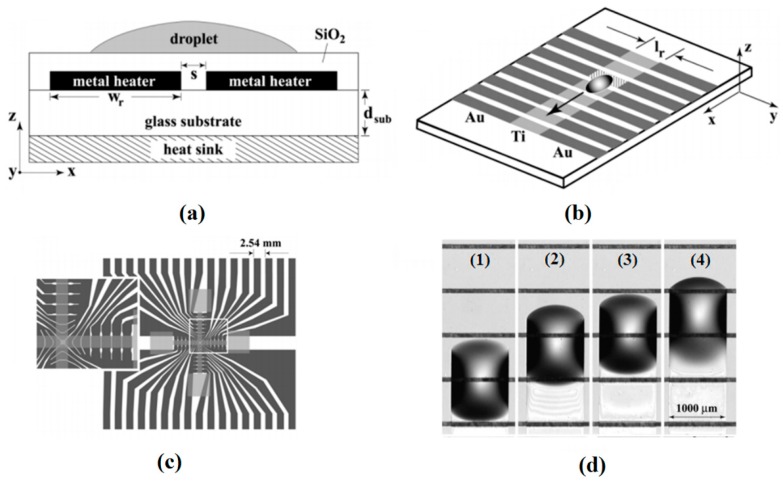
(**a**) Cross sectional view of a portion of the device containing two microheaters and an overlying droplet; (**b**) Top view of a liquid droplet moving along a liquophilic microstripe; (**c**) Top view of the resistor and contact layout; (**d**) A droplet moving along the heater array. The images sequence represent times (1) *t* = 0 s, (2) 44 s, (3) 88 s, and (4) 132 s. Copyright 2003, IEEE. Reprinted with permission from [190], A.A. Darhuber, J.P. Valentino, S.M. Troian, and S. Wagner, “Thermocapillary actuation of droplets on chemically patterned surfaces by programmable microheater arrays”, *Journal of Microelectromechanical Systems,* vol. 12, pp. 873–879, 2003.

Owing to progress in microfabrication, combination of embedded metal electrical resistors as microheaters and chemically patterned substrate surface led to a new microfluidic device for droplet and liquid stream actuation proposed by Darhuber and coworkers [190,191,192,193]. While local heating produces surface tension gradient, chemicals divided the substrate to liquiphobic and liquiphilic regions which has enhanced the liquid movement on the substrate. Figure 32 shows the proposed device. The array of Ti-resistors (shown in light gray) beneath the liquophilic stripes locally heat the droplet nearby modifying the surface tension and propelling the liquid toward the colder regions of the device surface. The dark gray stripes represent the leads and contacts (Au) for the heating resistors in Figure 32b. For a proof of concept device two intersecting liquophilic channels were connected to four liquophilic reservoir pads (Shown in semitransparent light gray) to build the resistor. The central region inside the white rectangle is magnified in the inset. They initiated the design by simulation to achieve the most efficient device [194].

**Figure 33:**
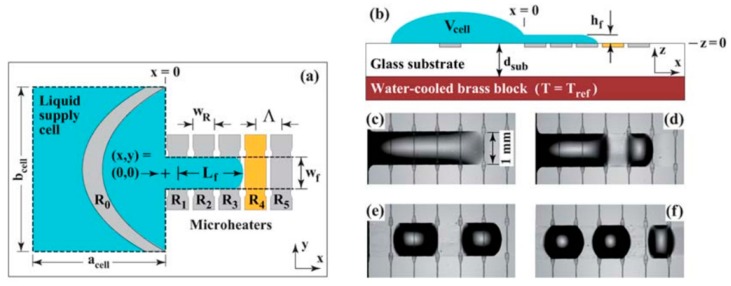
Top (**a**) and cross sectional (**b**) views of microfluidic device fabricated on glass substrate, (**c**) optical micrograph of a 1 mm wide liquid filament separated electronically into (**d**) one, (**e**) two, and (**f**) three droplets. Republished with permission of Royal Society of Chemistry, from [195], “Planar digital nanoliter dispensing system based on thermocapillary actuation”, A.A. Darhuber, J.P. Valentino, and S.M. Troian, *Lab on a Chip,* vol. 10, pp. 1061–1071, 2010; Permission conveyed through Copyright Clearance Center, Inc.

They also employed a similar heater pattern to use as a nano-dispenser [195]. As is shown in Figure 33, their device has been tested for droplet production with controlled size within the volume range of 10 to 500 nL. Similarly, Gao *et al.* proposed a device for actuation of drops on a solid surface [196]. They fabricated Ti/Au microheaters covered by silicon dioxide, and, by switching them sequentially, they succeeded to move silicone oil droplet on top.

**Figure 34:**
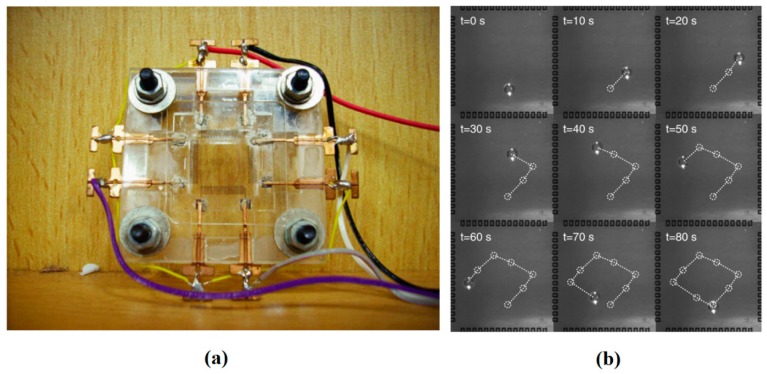
(**a**) The device proposed by Jiao *et al.* to produce a temperature field and actuate a confined droplet by changing the voltage supplied to the four heaters on four sides of the device. (**b**) Droplet migration for a specific heating code. Reproduced with permission from [198], Z. Jiao, X. Huang, and N.-T. Nguyen, “Manipulation of a droplet in a planar channel by periodic thermocapillary actuation”, *Journal of Micromechanics and Microengineering,* vol. 18, p. 045027, 2008. Copyright 2008, IOP Publishing, all rights reserved.

After fabrication of devices to manipulate drops inside microchannels or on solid substrates using coils or embedded heaters, thermocapillary-based microdevice industry evolved toward fabrication of path-free devices. Nguyen’s group showed the movement of a silicone oil drop in a square channel [197,198]. They designed a square enclosed glass channel with four Ti-Pt microheaters fabricated on four sides (Figure 34a). Owing to the thermocapillary forces, droplet movement was observed toward the least hot spot which could be anywhere on the substrate depending on the voltage applied to the heaters (the heating code). This observation is shown in Figure 34b. They also simulated the temperature field and correlated the temperature to the position of the drop through its surface tension.

**Figure 35:**
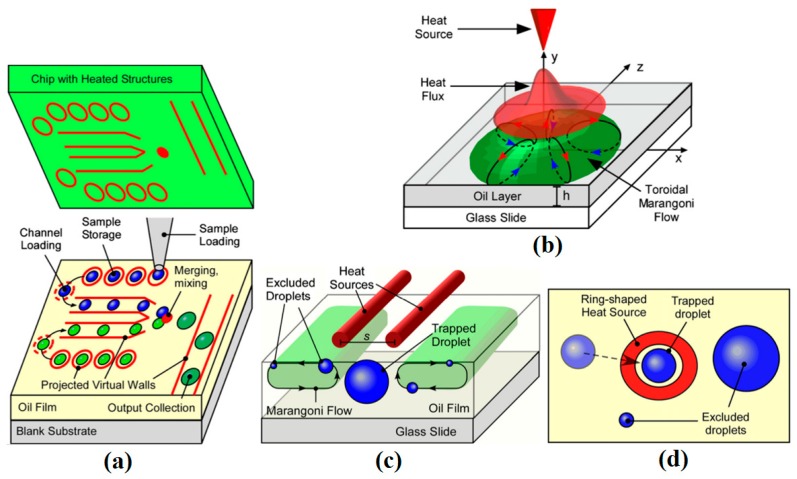
(**a**) Concept of a contactless microdroplet manipulator based upon Marangoni flows. The flow is driven by heat sources of various geometries suspended above the oil layer. The projected heat fluxes (shown in red) generate flows which emulate droplet channels, reservoirs, and mixers. (**b**) A microfluidic trap generated by a point heat source. Schematic is showing the suspended heat source, the projected Gaussian heat flux profile, and the toroidal flow region in the oil layer. (**c**) Virtual droplet channels generated by parallel linear heat sources. The channel boundaries are defined by the heat flux projected by two heated wires with separation *s* held parallel to the liquid surface. Target sized droplets are pulled into the channel by the subsurface flows. Orders are excluded. Marangoni flows are shown in green and with arrows. (**d**) Single droplet trapping with an annular heat flux. Schematic is showing the annular heat flux projected on the surface, the trapped droplet, and the exclusion of larger and smaller droplets. Reproduced with permission from [178], A.S. Basu and Y.B. Gianchandani, “Virtual microfluidic traps, filters, channels and pumps using Marangoni flows”, *Journal of Micromechanics and Microengineering,* vol. 18, p. 115031, 2008. Copyright 2008, IOP Publishing, all rights reserved.

At the same time, Basu and Gianchandani proposed a device to manipulate, trap, sort, and mix the droplets using thermocapillary effect produced by remote heaters on the free surface of a carrier liquid [178]. In their device, virtual channels, traps, filters, pumps, and valves are produced in the liquid film by fabricating different geometries of heat sources and suspending them just above the liquid surface in a non-contact manner. A point source can produce a mixer, an annular source is used for trapping, while two line sources are inducing virtual pumps in the liquid film (Figure 35).

**Figure 36:**
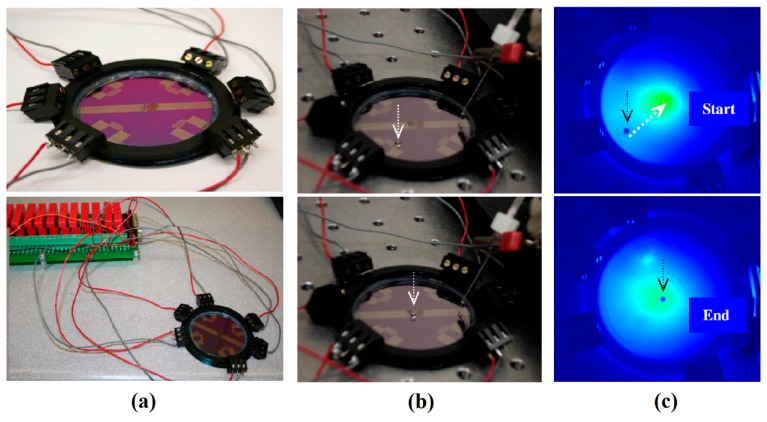
Fabricated device (**a**) for moving a levitated droplet of water on a film of FC-43 oil toward the hot spot where the underlying heater is switched on (**b**,**c**). Pictures (**c**) is taken by a FLIR SC5000 infrared thermal camera and shows the temperature gradient. Copyright 2011, IEEE. Reprinted and modified, with permission, from [182], E. Yakhshi-Tafti, R. Kumar, and H. Cho, “Thermally-actuated high speed droplet manipulation platform”, in Proceedings of 2011 16th International Solid-State Sensors, Actuators and Microsystems Conference (TRANSDUCERS), 2011, pp. 1484–1487.

Developing Marangoni flow inside a thin liquid film leads to a change of surface elevation and produces a depressed area in the hot region. On the other hand, it has been discussed that droplets stay levitated on the immiscible liquid films of different temperature under specific conditions of size and release height. Combining these two facts, our group proved the concept of dual behavior of the drops toward the temperature gradient based on their shape [181]. We also came up with a microdroplet actuator device on silicon substrate with fabricated Ti heaters. As is shown in Figure 36, the drops were actuated on top of the fluorocarbon oil (FC-43) using the same concept. Later on we proved that the shape of the droplet stays the same (spherical) and it is the state of equilibrium of the drop in relation to the liquid film that contributes to this dual behavior [184]. Droplets slip toward the hot spot as long as they stay levitated on top of the carrier liquid with a separating draining air film and once they get submerged they will be swept by the Marangoni flow. The dominant force on the droplets in the first stage is gravity while in the latter is drag. Convergence of levitated droplets to the hot spot is a novel idea for the controlled manipulation of droplets. Some of the known drawbacks of droplet actuation such as droplet pinning and hysteresis, and contamination were addressed with this finding.

### 6.2. Thermocapillary Valves, Switches, and Traps

**Figure 37:**
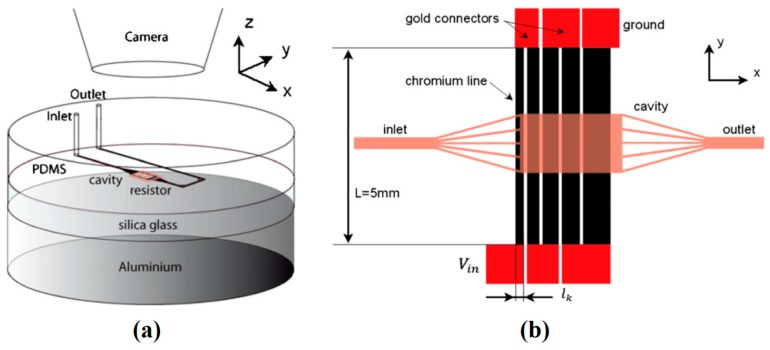
(**a**) Overview of the experimental device by Selva *et al.* (**b**) Top view of their experimental setup. The liquid fills the cavity positioned above the chromium resistors which have been placed in series and are connected by gold wires. Reproduced with permission from [200], B. Selva, J. Marchalot, and M.-C. Jullien, “An optimized resistor pattern for temperature gradient control in microfluidics”, *Journal of Micromechanics and Microengineering,* vol. 19, p. 065002, 2009. Copyright 2009, IOP Publishing, all rights reserved.

**Figure 38:**
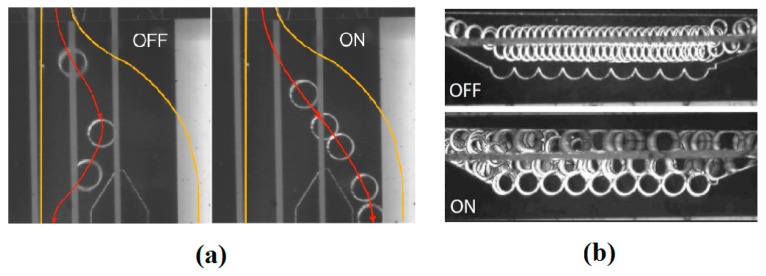
(**a**) Switching and (**b**) trapping of the droplets are shown using the device proposed by Selva *et al.* Republished with permission of Royal Society of Chemistry, from [199], “Thermocapillary actuation by optimized resistor pattern: bubbles and droplets displacing, switching and trapping”, B. Selva, V. Miralles, I. Cantat, and M.-C. Jullien, *Lab on a Chip,* vol. 10, pp. 1835–1840, 2010; Permission conveyed through Copyright Clearance Center, Inc.

Working on bubbles and droplets actuation in closed microfluidic platforms, Selva and co-workers proposed a method to increase the actuation velocity using Marangoni effect [199,200,201,202,203]. They designed an optimized heater pattern that can produce uniform temperature gradient along with variant cross section cavity to enhance elements (bubbles and droplets) manipulation. They reported the velocity of 1 cm/s and used their device for element displacement, switching, and trapping. The details of their device are depicted in Figure 37 and manipulation (switching and trapping) of droplets is shown in Figure 38.

**Figure 39:**
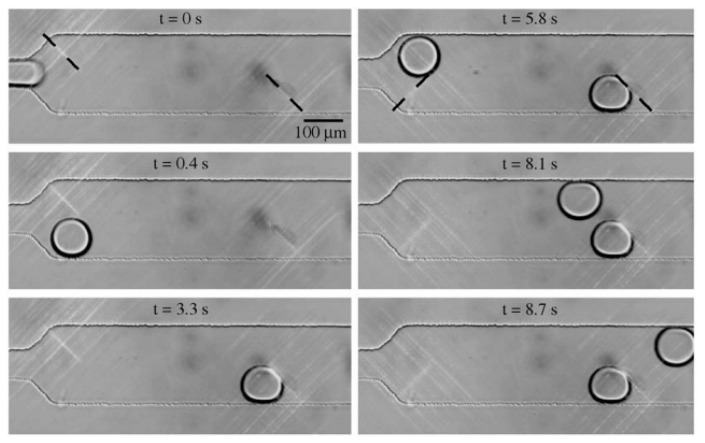
Image sequence showing how the drop order can be changed: The initial drop is sent down and held stationary, then successive droplets are sent up. The dashed lines overlay the position of laser patterns. Reprinted with permission from [204]. Copyright 2008, AIP Publishing LLC.

In an experimental effort, Cordero and coworkers proposed a new microfluidic device for thermocapillary manipulation of droplets based on holographic optical patterning [204]. Figure 39 shows how the drops were directed sequentially in their device. They showed that this scheme is more flexible than the conventional Gaussian routing and can enhance droplets controllability and manipulation speed.

**Figure 40:**
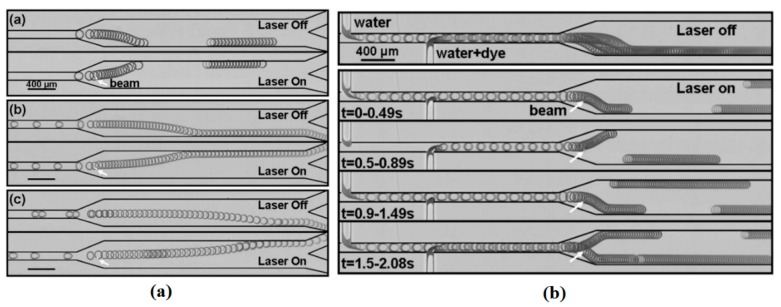
Both figures are superposition of successive snapshots to show the effect of laser heating on (**a**) droplet switching, and (**b**) droplet sorting. Reprinted with permission from [205]. Copyright 2008, AIP Publishing LLC.

De Saint Vincent and coworkers proposed a thermocapillary-based drop actuation, switching, and sorting device using laser beam to produce a hot spot [205]. Their device is claimed to be high throughput with drop velocities up to 1.3 cm/s and switching efficiency of 100%. Figure 40 shows switching and sorting of different kinds of droplets using point laser spot.

### 6.3. Thermocapillary Sensors

**Figure 41:**
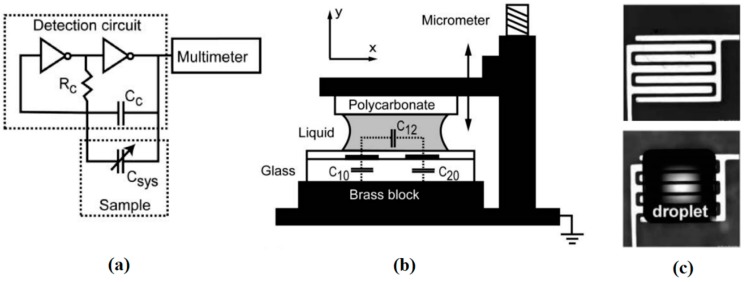
(**a**) Schematic diagram of a complete sensing circuit consisting of two inverters, one resistor *R*_c_, and one capacitor C_c_, which are attached in parallel to the system shown in (**b**) whose capacitance is given by *C*_sys_. Changes in *C*_sys_ detectable by the oscillation frequency shift are measured with an external multimeter. (**b**) Schematic diagram of the experimental setup containing the sensing electrode arrays. The liquid film thickness, *d*_liq_, is adjusted by vertical displacement of a (non-wetting polycarbonate sheet. The native electric field capacitance is denoted *C*_12_; the coupling capacitances with the brass block are labelled *C*_10_ and *C*_20_. (**c**) Top view of the actual device with and without droplet. Republished with permission of Royal Society of Chemistry, from [206], “Capacitive sensing of droplets for microfluidic devices based on thermocapillary actuation”, J.Z. Chen, A.A. Darhuber, S.M. Troian, and S. Wagner, *Lab on a Chip,* vol. 4, pp. 473–480, 2004; Permission conveyed through Copyright Clearance Center, Inc.

Thermocapillarity along with other techniques has been used over the years to fabricate microsensors. In 2004, a circuit was proposed by Chen *et al.* as shown in Figure 41a to measure the change of the sample (droplet) capacitance [206]. The droplet that is actuated to locate on top of the circuit on a glass substrate works as a capacity to connect the circuit. The metal electrodes fabricated at the bottom of the glass substrate also work as microheaters to move the droplets by inducing thermocapillary effect. This device is capable of sensing the droplet position, size, and composition by measuring its capacitance, and has a very short response time.

**Figure 42:**
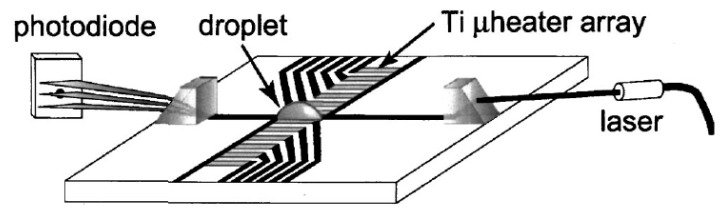
Schematic of the microdroplet detection and analysis proposed by Valentino *et al.* Reprinted with permission from [207]. Copyright 2005, AIP Publishing LLC.

One year later Valentino *et al.* designed a platform to actuate and mix the droplet, detect the droplet location and measure the mixing/reaction rate using a laser beam detector [207,208]. They used arrays of fabricated Ti microheaters to take advantage of the thermocapillary effect in manipulating and directing droplets to the measurement area. In this so called waveguide surface the laser beam is exposed trough the droplet and detection of droplet location, mixing, and reaction rate measurement becomes possible with the help of photodiode as shown in Figure 42. One of the mentioned applications of this device is in chromogenic and biological assay experiments.

**Figure 43:**
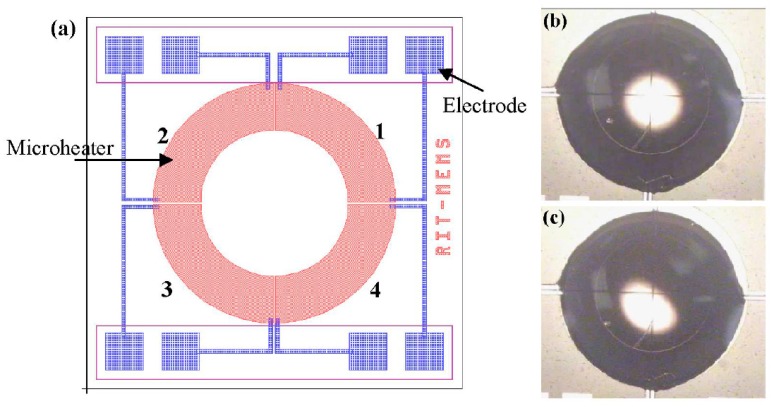
(**a**) The schematic of the device for micromirror actuation consisted of four quarter ring shaped heaters; (**b**) the device loaded with a droplet with a microplate on top. Switching the heaters leads to actuation of the droplet and hence tilting the microplate. Copyright 2009, IEEE. Reprinted, with permission, from [209], R. Dhull, I. Puchades, L. Fuller, and Y. Lu, “Optical Micromirror Actuation using Thermocapillary Effect in Microdroplets”, in Processings of IEEE 22nd International Conference on Micro Electro Mechanical Systems (MEMS 2009), 2009, pp. 995–998.

Dhull and coworkers proposed a method to actuate micromirrors by inducing thermocapillarity in a droplet on a solid surface [209]. The microplate has been placed on top of a microdroplet and voltage has been supplied to the quarter-ring-shaped heaters to produce temperature gradient between two sides of the droplet, inclining it toward cold side and hence tilting the microplate on top of it. They managed to produce up to 6.5° of tilt by supplying 30 V. Figure 43a depicts the schematic of the device, and the actual experiment is shown in Figure 43b. They have proposed that the microplate can be coated by a reflective material such as aluminum and be used as a micromirror.

Thermocapillary-based microdevices as discussed in this section can be categorized in general groups of pumps and actuators, mixers, valves, switches, and traps, and also sensors; although thermocapillarity has also been used in small scale for other purposes. Using Marangoni convection for manipulation of solid microbeads by Vela *et al.* [210], employing thermocapillarity for shaft work production by Hendarto and Gianchandani [211], and analog chemical computation by Lovass *et al.* [212] are some of these examples.

The path of development of thermocapillary-based microdevices -as is noticed in this section- started with actuation in microchannels and microtubes by coil heater. During the time with the progress in microfabrication, embedded heaters are replaced and actuation on open solid substrates have been under attention. This led to less drag and power consumption, and eventually more efficient devices. By entering open surface devices with liquid carrier films as substrates, issues such as contact angle hysteresis and pinning, cross contamination, and sample loss has also been addressed. Optical manipulation as the next step of this progress is widely in use these days for thermocapillary-based microdevices which has enhanced accuracy and efficiency furthermore although their drawback is lack of availability since focused laser beams are the necessary part of these types of devices. Researchers are still working on both physical and remote heaters to develop novel ideas.

## 7. Summary

Thermocapillarity as the effect of temperature-induced surface tension gradient on flow physics of the liquid, may lead to different forms of instabilities depending on the relative direction between temperature gradient to the liquid interface. If temperature gradient is perpendicular to the surface of the liquid, the thermocapillary instability is of the form of Marangoni convections and can be categorized into either linear or nonlinear steady basic states. Hydrothermal waves are produced and propagated in form of rolls with different directions and orientations for the cases that the temperature gradient is tangential to the interface. Surface tension gradient along the interface in these cases will lead to actuation.

The relation between thermocapillary instability and evaporation has been reported to be mutual, *i.e.,* each of them can induce or enhance the other one. According to the reports thermocapillarity affects the nature of evaporation which has been captured inside evaporating droplets. It can have a dual effect on the evaporation rate, and even sometimes may lead to condensation. While droplet spreading due to capillarity and gravity is enhancing evaporation owing to reduced drag force and increased exposed surface, Marangoni flow inside drops pushes the liquid to the peak of the drop and hence can be considered as a factor against evaporation. However, thermally-induced convection can lead to increasing the evaporation rate in some cases.

Thermocapillary-induced fluid flows inside drops and liquid films are used for device applications when bulk motion of liquid is needed. Different phenomena related to droplet-based thermocapillarity such as droplet spreading, bubble/droplet migration, mixing, and coalescence have been studied both experimentally and numerically. Droplets on solid substrates must be greater than a critical size to start moving under thermocapillarity and their velocity increases with temperature gradient while this causes the critical radius to decrease. On the other hand, the droplets that are too big or under an intense temperature gradient my split if they are on a solid substrate and deform due to reduction of surface tension if they are immersed in another liquid. Encapsulation of drops with surfactants or long chain alcohols can remedy this problem. A complicated phenomenon inside droplets is chaotic mixing which happens in laminar regime because of small length scales. There are two length scales involved in this behavior. The first one is related to the volume fraction of chaotic *versus* regular streamlines and the second one deals with the time required for homogenization of chaotic mixing. Besides increasing the temperature gradient, emission of switching laser beams with a frequency less than the response time of the liquid has been found as a way to increase mixing rate. Another interesting phenomenon is coalescence. For a droplet colliding a solid surface, collision leads to coalescence if the temperature of the droplet is less than that of the wall. Marangoni convection and hence formation of a draining film of air (or a second liquid in the immersed case) thicker than the van der Waals force range of the two fluids molecules stops the colliding droplet(s) from coalescence leading to a phenomenon named noncoalescence or nonwetting.

Taking advantage of aforementioned changes of fluid physics induced by thermocapillarity, numerous thermocapillary-based microfluidic devices have been proposed for different applications such as pumps and actuators, mixers, valves and switches, and also sensors. The heat sources in thermocapillary-based devices have been incorporated as either embedded or discrete remote elements. The first generation of these devices was proposed as closed systems in which fluidic medium was confined in microchannels or microtubes. Droplet actuation on solid surfaces using embedded configurations of metal heaters was later proposed owing to the progress in microfabrication techniques which led to more precise and efficient devices. Droplet pinning and contact angle hysteresis, sample contamination and evaporation, and limited velocity still remained as issues. Techniques such as levitated-droplet-on-liquid-film actuation and using laser beams as heat source were proposed to address these challenges to some level. Recently a unique dual-mode actuation based on the levitated and submerged droplet was observed and its practical implementation was demonstrated. All of this recent progress prompts renewed interest in thermocapillary phenomena in microfluidics for realization of microdevices that are efficient and accurate for chemical and biological applications. Table 2 summarizes thermocapillary-based microfluidic devices for various applications. 

**Table 2:** Selection of proposed thermocapillary-based microfluidic devices for various applications.

Configuration	Heat Source	Application	Refs.
Drops and Bubbles in Microchannels	Embedded Al	Droplet Actuation and Mixing	[187]
	Embedded Poly-Si	Thermocapillary Pumping (TCP)	[188]
	Embedded Cr/Ni	Bubble Micro-oscillator	[189]
	Embedded Cr	Droplet Switching, Sorting and Trapping	[199,200,201,202]
	Laser Beam	Droplet Manipulation	[204]
	Laser Beam	Droplet Switching and Sorting	[205]
Drops on Solid Surfaces	Embedded Ti/Au	Droplet Actuator	[190,191]
	Embedded Ti	Droplet Actuator	[207]
	Embedded Ti/Pt	Droplet Mixing/Reaction Measurement	[197,198]
	Embedded Poly-Si	Micromirror Actuator	[209]
	Embedded Au/Ti	Droplet Manipulation	[196]
	Embedded Ti/Au	Nano-Dispenser	[195]
	Embedded Ti/Au	Droplet Position, Size, Composition Measurement	[206]
Drops on Liquid Films	Suspended Heaters	Droplet Actuation, Mixing, Trapping, and Pumping	[178]
	Embedded Ti	Droplet Actuation, Mixing, Trapping, and Sorting	[182]
Miscellaneous	Laser Beam	Microbead Manipulation	[210]
	Suspended Heaters PCB	Mechanical Power Production	[211]
	Cold Metal	Chemical Computation	[212]
